# Regulatory KIR^+^RA^+^ T cells accumulate with age and are highly activated during viral respiratory disease

**DOI:** 10.1111/acel.13372

**Published:** 2021-05-27

**Authors:** Daan K. J. Pieren, Noortje A. M. Smits, Jeroen Hoeboer, Vinitha Kandiah, Rimke J. Postel, Rob Mariman, Josine van Beek, Debbie van Baarle, Jelle de Wit, Teun Guichelaar

**Affiliations:** ^1^ Centre for Infectious Disease Control National Institute for Public Health and the Environment Bilthoven The Netherlands; ^2^ Center for Translational Immunology University Medical Center Utrecht Utrecht University Utrecht The Netherlands

**Keywords:** aging, CD8^+^ T cells, killer‐cell immunoglobulin‐like receptors, regulatory T cells, respiratory viral infection, T‐cell activation, viral respiratory disease

## Abstract

Severe respiratory viral infectious diseases such as influenza and COVID‐19 especially affect the older population. This is partly ascribed to diminished CD8^+^ T‐cell responses a result of aging. The phenotypical diversity of the CD8^+^ T‐cell population has made it difficult to identify the impact of aging on CD8^+^ T‐cell subsets associated with diminished CD8^+^ T‐cell responses. Here we identify a novel human CD8^+^ T‐cell subset characterized by expression of Killer‐cell Immunoglobulin‐like Receptors (KIR^+^) and CD45RA (RA^+^). These KIR^+^RA^+^ T cells accumulated with age in the blood of healthy individuals (20–82 years of age, *n* = 50), expressed high levels of aging‐related markers of T‐cell regulation, and were functionally capable of suppressing proliferation of other CD8^+^ T cells. Moreover, KIR^+^RA^+^ T cells were a major T‐cell subset becoming activated in older adults suffering from an acute respiratory viral infection (*n* = 36), including coronavirus and influenza virus infection. In addition, older adults with influenza A infection showed that higher activation status of their KIR^+^RA^+^ T cells associated with longer duration of respiratory symptoms. Together, our data indicate that KIR^+^RA^+^ T cells are a unique human T‐cell subset with regulatory properties that may explain susceptibility to viral respiratory disease at old age.

## Graphical abstract

Viral infectious diseases such as influenza especially affect the older population due to immunological dysfunctions. We describe a new aging‐related subset of CD8^+^ T cells that is identified by the co‐expression of KIR and CD45RA and expresses features of senescence/exhaustion and immune suppression. This T‐cell subset increases with age and comprises a major part of the T cells that become activated during influenza correlating with disease in older persons, indicating clinical relevance.

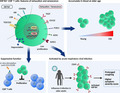

## 1 INTRODUCTION

Aging dramatically increases the risk for developing prolonged and more severe disease after acute viral respiratory infections (Fleming & Elliot, 2005; Jansen et al., 2007; McElhaney et al., 2016; Ruuskanen et al., 2011; Walsh et al., 2004). This has been shown for viruses such as influenza virus, respiratory syncytial virus (RSV), and coronaviruses (Jansen et al., 2007; Ruuskanen et al., 2011; Thompson et al., 2003; Walsh et al., 2004). Moreover, susceptibility of older adults for respiratory infections is currently being highlighted by the severe acute respiratory syndrome coronavirus 2 (SARS‐CoV‐2) pandemic (Phelan et al., 2020; Zhou et al., 2020). Aging‐related alterations may occur in many different parts of our immune system and collectively contribute to decreased host defense to infections resulting in risk for severe infectious disease. The risk of severe disease is in part explained by decreasing functionality of CD8^+^ T cells during the process of aging (Briceno et al., 2016; Decman et al., 2010, 2012; Lanna et al., 2017) resulting in impaired viral clearance (Graham et al., 1991; Slutter et al., 2013; Sridhar et al., 2013; Wang et al., 2015). To better understand the role of the impaired CD8^+^ T‐cell response during viral infection in the development of prolonged and severe disease in older adults, it is important to identify CD8^+^ T‐cell subsets that contribute to the decline of T‐cell‐mediated responses.

The decline of CD8^+^ T cell‐mediated immune responses with age is in part associated with changes in CD8^+^ T‐cell phenotype and defects in proliferation, often referred to as T‐cell exhaustion and/or senescence (reviewed in (Akbar & Henson, 2011; Akbar et al., 2016)). T‐cell exhaustion is characterized by increased expression of inhibitory receptors by CD8^+^ T cells (Wherry, 2011). In humans, expression of the co‐inhibitory receptor T‐cell immunoglobulin and ITIM domain (TIGIT) by CD8^+^ T cells has been shown to increase with age and is associated with diminished CD8^+^ T‐cell responses in the elderly (Song et al., 2018). Recent work demonstrated that effector memory (T_EM_) and terminally differentiated effector cells (T_EMRA_) were the most predominant CD8^+^ T‐cell subsets expressing TIGIT during aging (Song et al., 2018).

Recently, so‐called “virtual memory cells” have been identified in mice and humans (Jacomet et al., 2015; Quinn et al., 2018; White et al., 2016) as a new CD8^+^ T‐cell subset that accumulates with age (Chiu et al., 2013; Quinn et al., 2018; White et al., 2016). It is currently assumed that virtual memory cells are antigen‐naïve T cells that acquire a memory phenotype through homeostatic proliferation (Haluszczak et al., 2009; Sosinowski et al., 2013; White et al., 2016) and may act as bystander cells during infection (Haluszczak et al., 2009; White et al., 2016). In aged mice, virtual memory cells express a senescent phenotype with diminished capacity to participate in primary immune responses (Quinn et al., 2018). In humans, virtual memory CD8^+^ T cells are CD45RA^+^ cells that have been defined by expression of killer‐cell immunoglobulin‐like receptors (KIR) and/or the NK‐cell receptor NKG2A (Jacomet et al., 2015; Quinn et al., 2018). However, further phenotypical and functional characterization as well as the clinical relevance of these cells in humans during an ongoing viral infection is currently unknown. Moreover, although KIR^+^ and NKG2A^+^ cells have been regarded to collectively comprise one subset of virtual memory T cells, cells expressing either KIR or NKG2A may be different subsets. We therefore aimed to address the significance of KIR^+^ and NKG2A^+^ as individual subsets of CD45RA^+^ CD8^+^ T cells in aging by defining their phenotype, their functionality, and their relevance for respiratory viral infections in older adults.

Here we identified a human CD45RA^+^ subset that is characterized by expression of KIR and the lack of NKG2A as a unique CD8^+^ T‐cell subset. We provide evidence that these “KIR^+^RA^+^ CD8^+^ T cells” (KIR^+^ CD45RA^+^ NKG2A^−^ CD8^+^ T cells) accumulate with age and are therefore a previously unrecognized hallmark of aging. In contrast to virtual memory cells as previously described, we show that KIR^+^RA^+^ and NKG2A^+^RA^+^ CD8^+^ T cells are distinct subsets. KIR^+^RA^+^ T cells express high levels of the age‐related markers TIGIT and Helios, and appear to be a regulatory CD8^+^ T‐cell subset as we observed that these cells suppress the proliferation of other CD8^+^ T cells. Importantly, we show that KIR^+^RA^+^ T cells are activated during respiratory disease caused by viruses such as influenza viruses and coronaviruses, including SARS‐CoV‐2. Our data collectively show that KIR^+^RA^+^ cells accumulate within the CD8^+^ T‐cell population of older adults, become activated during respiratory viral infection, and provide evidence that activation of this T‐cell subset is a correlate of prolonged respiratory disease in influenza A virus‐infected older adults.

## 2 RESULTS

### 2.1 KIR^+^RA^+^ T cells accumulate with age, whereas NKG2A^+^RA^+^ T cells decline with age

**FIGURE 1:**
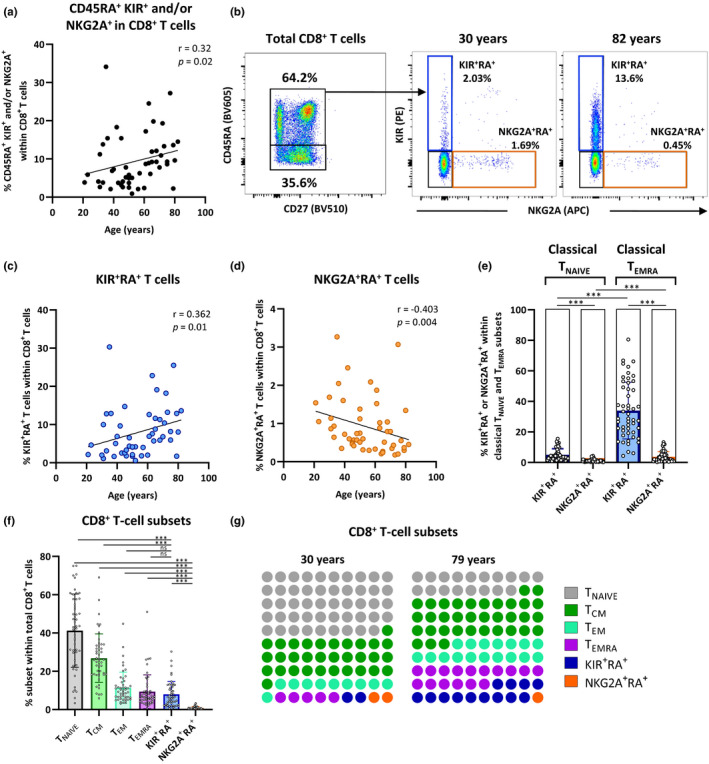
KIR^+^RA^+^ T cells accumulate with age, whereas NKG2A^+^RA^+^ T cells decline with age. KIR and NKG2A expression by CD45RA^+^ CD8^+^ T cells was analyzed in a cohort of healthy individuals ranging from 20–82 years of age (*n* = 50) using flow cytometry. (a) Relationship between the proportion of the virtual memory cell subset (CD45RA^+^ cells expressing KIR^+^ and/or NKG2A^+^) within the CD8^+^ T‐cell population and age. (b) Gating strategy to separate the virtual memory cell subset into KIR^+^NKG2A^−^ CD45RA^+^ CD8^+^ T cells (KIR^+^RA^+^ T cells, blue gate, % in total CD8^+^ T cells) and KIR^−^NKG2A^+^ CD45RA^+^ CD8^+^ T cells (NKG2A^+^RA^+^ T cells, orange gate). Relationship between (c) KIR^+^RA^+^ and (d) NKG2A^+^RA^+^ cell proportions within the total CD8^+^ T‐cell population and age. (e) Proportions of KIR^+^RA^+^ and NKG2A^+^RA^+^ T cells within the classically defined T_NAIVE_ (CD45RA^+^CD27^+^CD8^+^ T cells) and T_EMRA_ (CD45RA^+^CD27^−^CD8^+^ T cells) subsets. (f) Proportions of the six CD8^+^ T‐cell subsets among the CD8^+^ T‐cell population. (g) The proportional change with age of each cell subset within the CD8^+^ T‐cell population of representative individuals at young and older age. Correlations (*r* values) were assessed by Spearman test. Statistical significance of data presented in the bar graphs (means ± *SD*) (e, f) was determined using Friedman test (with Dunn’s post‐test) (***p < 0.001)

Virtual memory cells identified as CD45RA^+^ CD8^+^ T cells expressing KIR and/or NKG2A accumulate with age in Peripheral blood mononuclear cells (PBMCs) of healthy individuals (*n* = 50) (Figure 1a), which is in line with previous reports (Jacomet et al., 2015; Quinn et al., 2018; White et al., 2016). However, the accumulation of CD45RA^+^ CD8^+^ T cells positive for KIR or NKG2A differed between young and aged individuals (Figure 1b, Figure S1a for gating strategy). We observed an increase in the proportion of KIR^+^ NKG2A^−^ cells with age (Figure 1c), whereas the proportion of KIR^−^ NKG2A^+^ cells decreased with age (Figure 1d). CD45RA^+^ KIR^+^NKG2A^−^ cells were abundantly present, on average 30%, within the classically defined terminally differentiated effector cell subset (T_EMRA_) defined by CD27^−^ and CD45RA^+^ (Figure 1e). Based on these findings we hypothesize that the so‐called “virtual memory cell” subset comprises two different subsets; KIR^+^ NKG2A^−^ CD45RA^+^ T cells and KIR^−^ NKG2A^+^ CD45RA^+^ T cells, which we will conveniently address as KIR^+^RA^+^ T cells and NKG2A^+^RA^+^ T cells, respectively.

By regarding KIR^+^RA^+^ T cells and NKG2A^+^RA^+^ T cells as separate CD8^+^ T‐cell subsets, the CD8^+^ T‐cell population can be divided into six different subsets (Figure 1f, Figure S1a): naive T cells (T_NAIVE_, CD45RA^+^CD27^+^KIR^−^NKG2A^−^), central memory cells (T_CM_, CD45RA^−^CD27^+^), effector memory cells (T_EM_, CD45RA^−^CD27^−^), T_EMRA_ cells (CD45RA^+^CD27^−^KIR^−^NKG2A^−^), KIR^+^RA^+^ cells (CD45RA^+^KIR^+^NKG2A^−^), and NKG2A^+^RA^+^ cells (CD45RA^+^KIR^−^NKG2A^+^). Of these subsets, the proportions of T_NAIVE_ and NKG2A^+^RA^+^ T cells decline with age, the proportion of T_CM_ remains stable, whereas the T_EM_, T_EMRA_, and KIR^+^RA^+^ T cells increase with age (Figure 1g, Figure S1b). Thus, different proportions of KIR^+^RA^+^ and NKG2A^+^RA^+^ T cells with progressing age may indicate that these are distinct cell subsets.

### 2.2 The gene expression profile of KIR^+^RA^+^ T cells is distinct from that of NKG2A^+^RA^+^ T cells and shows features of aging and regulation

**FIGURE 2:**
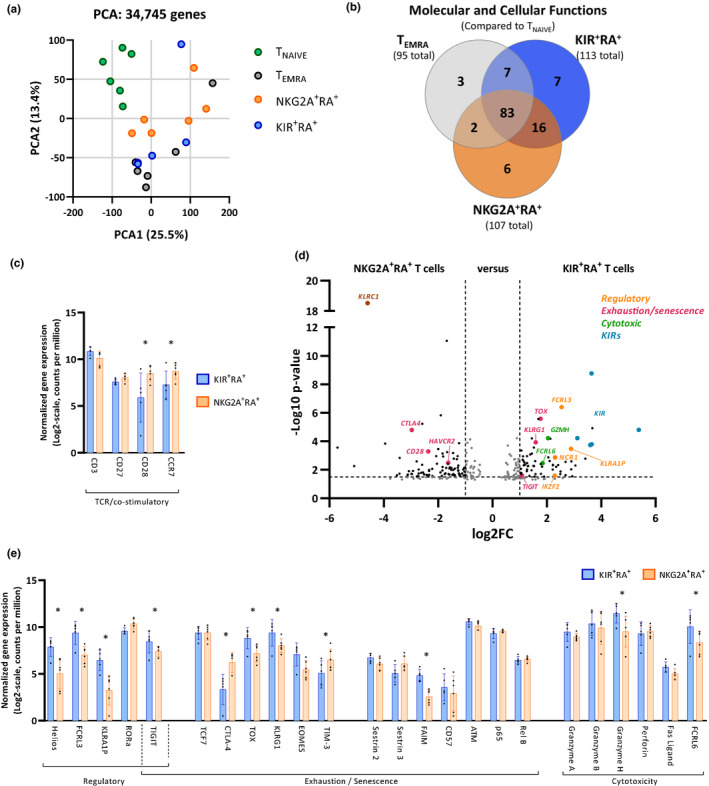
The gene expression profile of KIR^+^RA^+^ T cells is distinct from that of NKG2A^+^RA^+^ T cells and shows features of aging and regulation. KIR^+^RA^+^, NKG2A^+^RA^+^, T_NAIVE_, and T_EMRA_ cell subsets were sorted from healthy donor PBMCs (*n* = 6) and their gene expression profiles were analyzed by RNA sequencing. (a) Unsupervised principal component analysis (PCA) on the total transcriptome (34,745 genes) showing clustering of the four sorted cell subsets along components 1 and 2. (b) Venn diagram showing the unique and shared molecular and cellular functions for each of the three cell subsets compared to T_NAIVE_ cells as identified by ingenuity pathway analysis (IPA). (c) Comparison of detected transcripts associated with TCR‐ and co‐stimulation (shown by the names of the proteins they encode) between KIR^+^RA^+^ T cells and NKG2A^+^RA^+^ T cells. (d) Volcano plot indicating differences in gene expression between NKG2A^+^RA^+^ and KIR^+^RA^+^ T cells. The different colors indicate genes related to regulation, exhaustion/senescence, cytotoxicity, and KIR receptors. (e) Comparison of expression of selected genes associated with cellular regulation, exhaustion/senescence, and cytotoxicity between KIR^+^RA^+^ T cells and NKG2A^+^RA^+^ T cells. For each of these analyses, an absolute log2 Fold Change >1.0; −Log10 *p*‐value of >1.5 corresponding to *p* < 0.05 at a false discovery rate (FDR) of 0.10 was used

To further understand differences between KIR^+^RA^+^ and NKG2A^+^ T cells, we sorted these cell subsets (Figure S2a) and analyzed their gene expression profiles by RNA sequencing (RNA‐seq). As a reference, we included T_NAÏVE_ and T_EMRA_ cells in this analysis (Figure S2b). Unsupervised principal component analysis (PCA) on the total transcriptome (34,745 genes) reveals that T_NAIVE_ cells are distinct from the other three cell subsets (Figure 2a), but also reveals differences between KIR^+^RA^+^, NKG2A^+^RA^+^, and T_EMRA_ cells. In addition, the PCA indicates that the variation among these memory subsets is highly donor‐dependent (Figure S2c).

To asses if KIR^+^RA^+^ and NKG2A^+^RA^+^ T cells may have acquired distinct molecular and cellular functions, we first identified differentially expressed genes (DEGs) between the KIR^+^RA^+^, NKG2A^+^RA^+^, T_EMRA_, and T_NAIVE_ cells using a paired analysis (matched donor pairs). These DEGs were used to identify enrichment of unique pathways of molecular and cellular functions in KIR^+^RA^+^, NKG2A^+^RA^+^, and T_EMRA_ cells that discriminate these memory subsets from T_NAIVE_ cells (Figure S3). Besides 83 pathways shared by these three memory subsets, sixteen pathways are shared between KIR^+^RA^+^ and NKG2A^+^RA^+^ cells but absent in T_EMRA_ cells, which are mainly linked to chemotaxis and recruitment of cells (Figure 2b; Figure S3). KIR^+^RA^+^ T cells are uniquely enriched for seven pathways (Figure 2b), including pathways related to killing of lymphocytes and mononuclear leukocytes (Figure S3), whereas NKG2A^+^RA^+^ T cells are enriched for six unique pathways, associated with innate antigen presenting cell features (Figure S3). These findings indicate that both KIR^+^RA^+^ and NKG2A^+^RA^+^ T cells have acquired distinct molecular and cellular functions.

As numbers of KIR^+^RA^+^ T cells increase with age (Figure 1c,d), we focused our analyses on the characterization of this cell subset. Compared to NKG2A^+^RA^+^ T cells, genes involved in co‐stimulation (*CD28*, *CCR7*) are downregulated in KIR^+^RA^+^ T cells (Figure 2c). In addition, KIR^+^RA^+^ T cells showed enrichment for transcripts related to a regulatory cell phenotype, such as *IKZF2* (encoding Helios) (Kim et al., 2015), *FCRL3* (Nagata et al., 2009), and *KLRA1P* (Ly46 in mice) (Kim et al., 2011) (Figure 2d,e). Moreover, the transcriptome of KIR^+^RA^+^ T cells was enriched for several exhaustion/senescence‐related transcripts (*TIGIT*, *TOX*, *KLRG1*, and decreased *CD28*) (Bengsch et al., 2010) and transcripts of the senescence‐related anti‐apoptotic gene *FAIM* (Figure 2d,e), which may indicate cellular senescence (Huo et al., 2010), although other senescence‐associated genes (e.g., *SESN2*, *SESN3*, *B3GAT1*, *ATM*, *RELA*, *RELB*, *BCL2*) were not differentially expressed. Thus, our transcriptome analysis shows that the transcriptome of KIR^+^RA^+^ T cells is enriched for aging‐ and regulatory‐associated genes, which distinguishes these cells from NKG2A^+^RA^+^ T cells that had previously been regarded to be part of the same CD8^+^ T‐cell subset.

### 2.3 TIGIT^Hi^CD226^Low^ KIR^+^RA^+^ T cells contribute to age‐related TIGIT expression in CD8^+^ T cells

**FIGURE 3:**
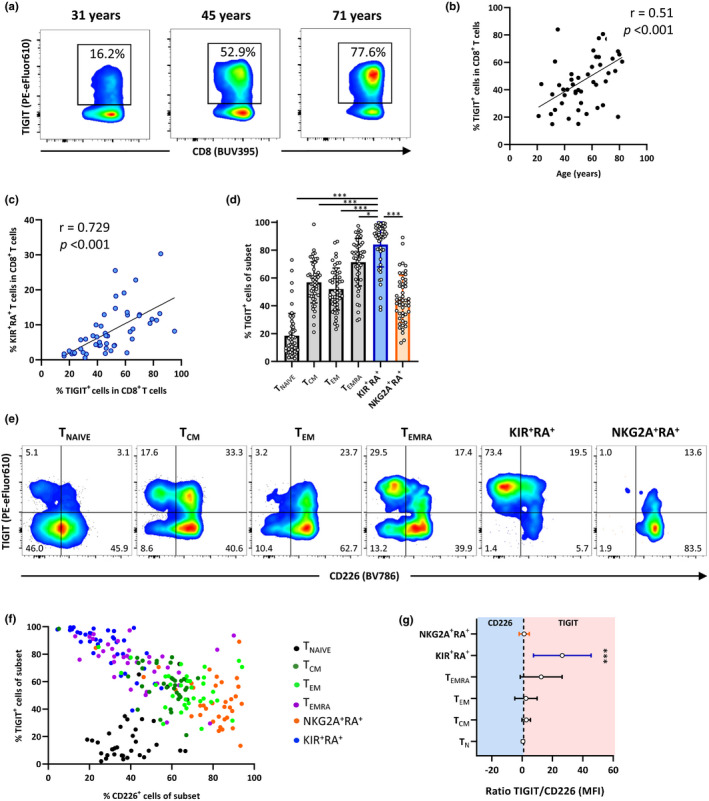
TIGIT^Hi^CD226^Low^ KIR^+^RA^+^ T cells contribute to age‐related TIGIT expression in CD8^+^ T cells. (a) Flow cytometry plots show TIGIT expression by total CD8^+^ T cells in selected individuals of different ages. (b) Relationship between the frequency of TIGIT^+^ cells within the total CD8^+^ T‐cell population and age in healthy individuals (*n* = 50). (c) Relationship between the frequency of KIR^+^RA^+^ cells and the frequency of TIGIT^+^ cells within the CD8^+^ T‐cell population. (d) The frequency TIGIT^+^ cells within the indicated CD8^+^ T‐cell subsets. (e) Representative flow cytometry plots of TIGIT and CD226 expression within each of the six CD8^+^ T‐cell subsets. (f) Relationship between TIGIT^+^ and CD226^+^ cell frequencies for each cell subset, as well as (g) the ratio between TIGIT and CD226 per cell subset based on the median expression per cell (MFI) of these markers. Correlations (*r* values) were assessed by Spearman test. Statistical significance of data presented in the bar graph (means ±*SD*) was determined using Friedman test (with Dunn’s post‐test). (*p < 0.05, **p < 0.01, ***p < 0.001)

The co‐inhibitory molecule TIGIT was one of the molecules that we identified in the transcriptome of KIR^+^RA^+^ T cells and has previously been described as a marker for regulation (Fuhrman et al., 2015; Joller et al., 2014), exhaustion (Chew et al., 2016; Johnston et al., 2014), and has recently been linked to aging (Song et al., 2018). We therefore questioned whether the accumulation of KIR^+^RA^+^ T cells may be linked to the aging‐related increase in TIGIT^+^ CD8^+^ T cells. Consistent with previous findings (Song et al., 2018), the frequency of TIGIT^+^ cells of total CD8^+^ T cells increased with age (Figure 3a,b). Strikingly, the proportion of KIR^+^RA^+^ T cells showed a strong positive correlation with the frequency of TIGIT^+^ CD8^+^ T cells (Figure 3c). Among all memory T‐cell subsets in our study, this correlation was strongest for the KIR^+^RA^+^ T cells (Figure S4a). KIR^+^RA^+^ T cells indeed showed the highest frequency of TIGIT^+^ cells and median expression per cell (MFI) compared to all other CD8^+^ T‐cell subsets (Figure 3d, Figure S4b). These findings show that KIR^+^RA^+^ T cells contribute to the accumulation of TIGIT^+^ CD8^+^ T cells with age. In addition, expansion of TIGIT^+^ CD8^+^ T cells can be multifactorial as TIGIT is also expressed by TEMRA and TEM cell subsets, which are found to expand with aging (Figure S1b).

The co‐stimulatory receptor CD226 competes with TIGIT for interaction with their shared ligands (Johnston et al., 2014). The ratio between CD226 and TIGIT on a CD8^+^ T cell determines the threshold for its proliferative and activation potential (Cella et al., 2010; Chew et al., 2016; Johnston et al., 2014; Tauriainen et al., 2017). Combining analyses of TIGIT and CD226 expression resulted in a separation of the six T‐cell subsets (Figure 3e). KIR^+^RA^+^ T cells expressed the highest frequency of TIGIT^+^ cells and the lowest frequency of CD226^+^ cells, and clearly separated from NKG2A^+^RA^+^ T cells based on these two markers (Figure 3f). The ratio between expression of TIGIT and CD266 by KIR^+^RA^+^ T cells showed that the balance between these markers is strongly skewed toward TIGIT in this subset (Figure 3g). Thus, KIR^+^RA^+^ T cells are characterized by TIGIT^Hi^CD226^Low^ expression and contribute to age‐related TIGIT expression.

### 2.4 KIR^+^RA^+^ T cells are responsive to stimulation but have low proliferative capacity

**FIGURE 4:**
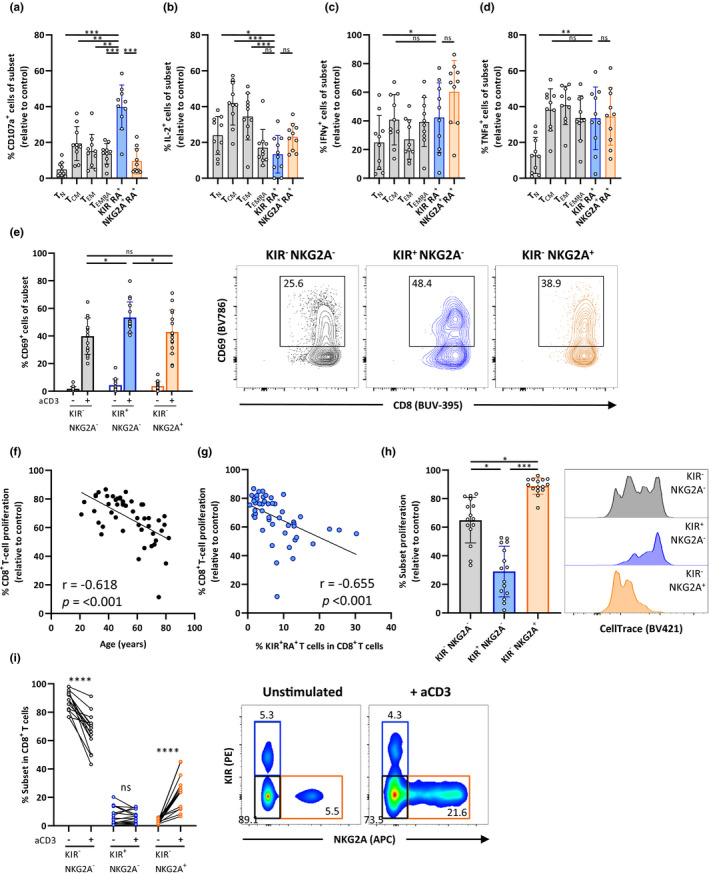
KIR^+^RA^+^ T cells are responsive to stimulation but have low proliferative capacity. Accumulation of intracellular cytokines was measured after exposure of total PBMCs of healthy individuals (*n* = 10) to PMA/Ionomycin for 6 hr. Frequency of (a) CD107a^+^, (b) IL‐2^+^, (c) IFN‐γ^+^, and (d) TNF‐α^+^ cells within the indicated CD8^+^ T‐cell subsets (relative to medium control as calculated by % positive cells cultured with PMA/ionomycin minus % positive cells in medium control). (e) PBMCs of healthy individuals (*n* = 15) were cultured for 3 days with (+) or without (−) stimulatory anti‐CD3, after which the frequency of CD69^+^ cells was determined in CD8^+^ T cells that were KIR^−^NKG2A^−^, KIR^+^NKG2A^−^, or KIR^−^NKG2A^+^ as shown in the bar graph and representative flow cytometry plots. CellTrace‐labeled PBMCs of healthy individuals (*n* = 50) were cultured with stimulatory anti‐CD3 for 3 days to detect proliferation of CD8^+^ T cells. Relationship between the frequency of proliferated CD8^+^ T cells (relative to medium control) and (f) age, and (g) ex vivo frequency of KIR^+^RA^+^ cells within the total CD8^+^ T‐cell population. (h) Proliferation of CD8^+^ T cells that are KIR^−^NKG2A^−^, KIR^+^NKG2A^−^, or KIR^−^NKG2A^+^ and the representative flow cytometry plot (*n* = 15). (I) Proportion and representative flow cytometry histograms of KIR^−^NKG2A^−^, KIR^+^NKG2A^−^, and KIR^−^NKG2A^+^ cells within the CD8^+^ T‐cell population cultured with (+) or without (−) stimulatory anti‐CD3 (*n* = 15). Correlations (*r* values) were assessed by Spearman test. Statistical significance of data presented in bar graphs (means ± *SD*) was determined using row‐matched one‐way ANOVA (with Geisser‐Greenhouse correction and Dunnett’s post‐test) (a–d), Friedman test (with Dunn’s post‐test) (e, h), or Wilcoxon matched‐pairs test (i). (*p < 0.05, **p < 0.01, ***p < 0.001, ****p < 0.0001, ns = not significant)

We next characterized the functionality of KIR^+^RA^+^ T cells. Upon non‐specific stimulation with PMA/ionomycin, KIR^+^RA^+^ T cells highly expressed CD107a compared to the other cell subsets, suggesting enhanced cellular degranulation (Figure 4a). The frequency of KIR^+^RA^+^ T cells producing IL‐2 was similar to levels found in T_EMRA_ and NKG2A^+^RA^+^ T cells (Figure 4b). Frequencies of KIR^+^RA^+^ T cells producing IFN‐γ and TNF‐α or combination of these cytokines, did not significantly differ from those found in the other subsets (Figure 4c,d, Figure S5a). These findings show that the KIR^+^RA^+^ subset is not hampered in production of cytokines.

We further assessed responsiveness of the KIR^+^RA^+^ T cells by assessing their capacity to upregulate the activation marker CD69 and proliferate in response to anti‐CD3 stimulation of PBMCs for 3 days. CD8^+^ T cells expressing KIR showed the highest frequency of CD69^+^ cells compared to cells that expressed NKG2A or cells that did not express KIR or NKG2A (Figure 4e). However, sorted KIR^+^RA^+^ T cells did not show increased frequencies of CD69^+^ cells compared to sorted KIR^−^NKG2A^−^ and NKG2A^+^RA^+^ T cells (Figure S5b). This suggests that KIR^+^RA^+^ T cells may depend on other cells and/or cell‐derived cytokines for their activation. In support of this notion, we found that of KIR^+^RA^+^ T cells become activated by the presence of exogenous T‐cell stimulating cytokines interleukin‐2 or interleukin‐15, or by soluble factors secreted by PBMC in response to CD3/CD28‐coupled beads, instead of anti‐CD3 (Figure S5c). Furthermore, we observed under the conditions we tested that the proliferative capacity of the overall CD8^+^ T‐cell population declined with age (Figure 4f). When we analyzed correlations of CD8^+^ T‐cell proliferation with proportions of the different T‐cell subsets (Figure 4g and Figure S5d), we found that this overall decline of proliferation strongly correlated with increased proportions of KIR^+^RA^+^ T cells present ex vivo (Figure 4g). This negative correlation may be explained by non‐proliferative KIR^+^RA^+^ T cells, despite their higher activation status. We therefore assessed proliferation in KIR^+^NKG2A^−^ and the KIR^−^ cell populations after anti‐CD3‐mediated stimulation of PBMCs. CD8^+^ T cells expressing KIR showed the lowest proliferation compared to the populations that did not express KIR (Figure 4h), which appears not to be restricted to old age (Figure S5e). This suggests that the correlation between decreased CD8^+^ T‐cell proliferation and increased proportion of KIR^+^RA^+^ T cells can be explained by low proliferative capacity of KIR^+^RA^+^ T cells. Indeed, the proportion of cells that were KIR^+^ did not expand whereas the proportion of cells that expressed NKG2A did (Figure 4i). Together, these findings show that despite high activation potential of KIR^+^RA^+^ T cells, KIR^+^RA^+^ T cells have low proliferative capacity. Since the proportion of KIR^+^RA^+^ T cells among the total CD8^+^ T‐cell pool accumulates with age, their lack of proliferative capacity may partly contribute to the decreased proliferation of the total CD8^+^ T‐cell pool at older age.

### 2.5 KIR^+^RA^+^ T cells show a distinct regulatory CD8^+^ T‐cell phenotype and suppress proliferation of KIR^−^NKG2A^−^ CD8^+^ T cells

**FIGURE 5:**
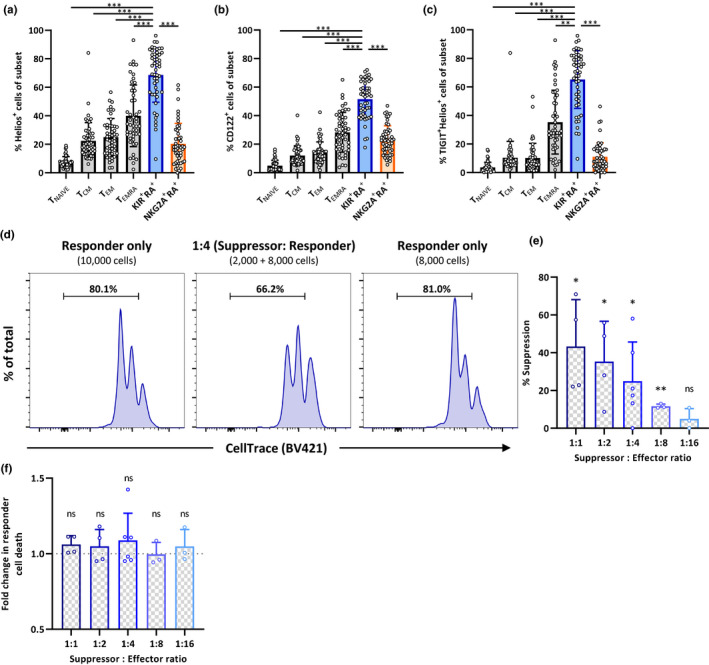
KIR^+^RA^+^ T cells show a distinct regulatory CD8^+^ T‐cell phenotype and suppress proliferation of KIR^−^ NKG2A^−^ CD8^+^ T cells. Frequency of (a) Helios^+^, (b) CD122^+^, and (c) TIGIT^+^Helios^+^ cells within the indicated CD8^+^ T‐cell subsets of healthy individuals (*n* = 50). For suppression assays, suppressor cells (KIR^+^RA^+^ T cells) and responder cells (KIR^−^NKG2A^−^ T cells) were sorted, added in different dose‐range ratios ranging from 1:1 to 1:16 (suppressor:responder), and cultured with stimulatory anti‐CD3 for 3 days. (d) Representative flow cytometry histograms show the frequency of proliferated KIR^−^NKG2A^−^ CD8^+^ T cells in responder only conditions (10,000 and 8,000 cells/well) and in a 1:4 suppressor:responder ratio. (e) Percentage of suppression and (f) fold change in responder cell death derived from suppression assays performed in *n* = 3–6 individual assays per indicated suppressor:responder ratio. Statistical significance of data presented in bar graphs (means ± *SD*) was determined using Friedman test (with Dunn’s post‐test) (a–c), or paired *t* test (e,f). (*p < 0.05, **p < 0.01, ***p < 0.001, ns = not significant)

The negative correlation between the proportion of KIR^+^RA^+^ T cells and CD8^+^ T‐cell proliferation may also imply that KIR^+^RA^+^ T cells regulate proliferation of other CD8^+^ T cells. TIGIT and Helios are markers for CD4^+^ regulatory T cells (T_REGS_) (Fuhrman et al., 2015; Kim et al., 2015; Thornton et al., 2010). Since KIR^+^RA^+^ T cells highly express TIGIT at the cell surface and our RNA‐seq data indicate enhanced TIGIT and expression of Helios transcripts, we explored the possibility that KIR^+^RA^+^ T cells might be regulatory CD8^+^ T cells. We first confirmed the regulatory phenotype of KIR^+^RA^+^ T cells by high intracellular expression of Helios protein by flowcytometry (Figure 5a) as well as by high cell surface expression of the CD8^+^ T_REG_‐associated marker CD122 (Dai et al., 2010) (Figure 5b). KIR^+^RA^+^ T cells showed the highest frequency of Helios^+^ and CD122^+^ cells compared to all other CD8^+^ cell subsets (Figure 5a,b), as well as expression of these markers per cell (Figure S6a,b). Combined expression of TIGIT and Helios even further delineated KIR^+^RA^+^ T cells from all other subsets (Figure 5c), indicating a distinct regulatory T‐cell phenotype of KIR^+^RA^+^ T cells.

We next investigated whether KIR^+^RA^+^ T cells could functionally regulate T‐cell proliferation in a suppression assay. Addition of KIR^+^RA^+^ T cells reduced proliferation of responder T cells (KIR^−^ NKG2A^−^ CD8^+^ T cells) (Figure 5d) in a dose‐dependent manner (Figure 5e). Adding responder cells instead of KIR^+^RA^+^ T cells to the responder cells did not reduce their proliferation, indicating suppression by KIR^+^RA^+^ T cells (Figure 5d left and right panels). Together, we show that KIR^+^RA^+^ T cells are a CD8^+^ T‐cell subset with regulatory phenotype and suppressive capacity. Therefore, accumulation of KIR^+^RA^+^ T cells with age may suggest that these cells contribute to suppressing immunity in older adults.

Killing of effector T cells may be a mechanism by which CD8^+^ T cells could mediate suppression. However, when analyzing the number of dead cells among T cells in our suppression assays, we did not observe increased cell dead in the presence of KIR^+^RA^+^ T cells (Figure 5f).

### 2.6 KIR^+^RA^+^ T cells are highly activated during acute respiratory infection in older adults as bystander cells

To investigate whether KIR^+^RA^+^ T cells play a role in dampening protection in the elderly, we analyzed the presence and activation status of KIR^+^RA^+^ T cells in peripheral blood of older adults (62–83 years of age, *n* = 36) suffering from a common respiratory viral infection (Table S1) (van Beek et al., 2017). Blood samples were obtained at the acute phase of infection (within 3 days of fever onset) and during follow‐up at 2 and 8 weeks.

**FIGURE 6:**
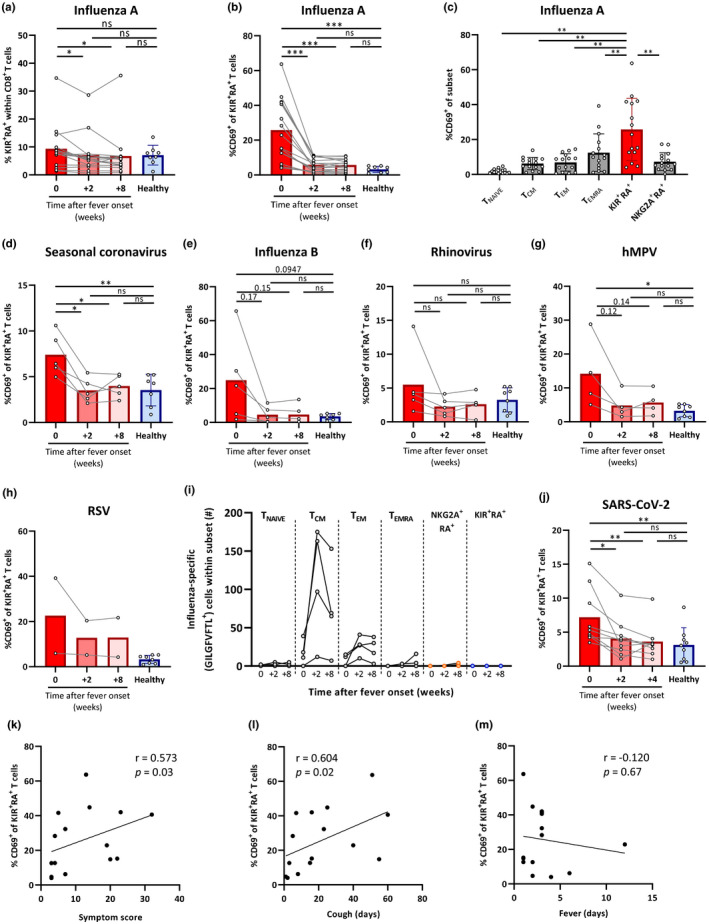
KIR^+^RA^+^ T cells are highly activated during acute respiratory infection in older adults as bystander cells and correlate with prolonged disease. Blood samples from older adults suffering from a common respiratory virus infection (62–83 years of age, *n* = 36) were analyzed at the acute phase (0) (within 2 days of fever onset), and during follow‐up at +2 and +8 weeks. Healthy asymptomatic individuals (61–82 years of age, *n* = 8) from the same cohort were used as control samples. (a) Frequency of KIR^+^RA^+^ T cells within the CD8^+^ T‐cell population and (b) the frequency of CD69^+^ cells in KIR^+^RA^+^ T cells at the three time points in influenza A‐infected older adults (*n* = 15) and healthy asymptomatic controls (*n* = 8). (c) The frequency of CD69^+^ cells in six different CD8^+^ T‐cell subsets. The frequency of CD69^+^ in KIR^+^RA^+^ T cells in the blood of older adults infected with (d) a seasonal coronavirus (*n* = 5), (e) influenza B virus (*n* = 5), (f) Rhinovirus (*n* = 5), (g) human metapneumovirus (hMPV) (*n* = 4), or (h) respiratory syncytial virus (RSV) (*n* = 2). (i) The number of CD8^+^ T cells specific for the immunodominant Influenza A matrix protein epitope GILGFVFTL detected by flow cytometry over time in the indicated CD8^+^ T‐cell subsets of HLA‐A*02^+^ influenza A‐infected individuals (*n* = 4). (j) The frequency of CD69^+^ cells in KIR^+^RA^+^ T cells in the blood of adults infected with SARS‐CoV‐2 and suffering from COVID‐19 (*n* = 9). Relationship between the frequency of CD69^+^ cells within the KIR^+^RA^+^ T‐cell subset and (k) the calculated symptom score, (l) duration of cough, and (m) duration of fever in older adults suffering from influenza A virus infection (*n* = 15). Correlations (*r* values) were assessed by Spearman test. Statistical significance of data presented in bar graphs (mean ± *SD*) was determined using row‐matched one‐way ANOVA (with Geisser‐Greenhouse correction and Dunnett’s post‐test) for the difference between the time points and Mann–Whitney *U* test was used to determine the difference between infected and asymptomatic healthy individuals (a, b, d–g, j). Row‐matched one‐way ANOVA (with Geisser‐Greenhouse correction and Dunnett’s post‐test) was used in (c). (*p < 0.05, **p < 0.01, ***p < 0.001, ns = not significant, or the exact *p*‐value is shown.)

The proportion of KIR^+^RA^+^ T cells within the total CD8^+^ T‐cell pool remained relatively stable from the acute phase throughout the following eight weeks, as exemplified in influenza A virus‐infected individuals (Figure 6a) and also by other viral infections in older adults (Figure S7a). However, we observed an increased frequency of activated CD69^+^ KIR^+^RA^+^ T cells in older adults during the acute phase of influenza A virus infection compared to the follow‐up at two and eight weeks, and compared to healthy older adults (Figure 6b, Figure S7b). At the acute phase of influenza A virus infection, KIR^+^RA^+^ T cells showed the highest frequencies of CD69^+^ cells compared to all other CD8^+^ T cells defined in our analyses (Figure 6c, Figure S7c). Significant activation of KIR^+^RA^+^ cells was also found for individuals infected with a seasonal coronavirus (Figure 6d) and similar trends were found for other respiratory viruses included in the study (Figure 6e–h).

To study whether KIR^+^RA^+^ T cells are virus antigen‐specific T cells, we assessed influenza specificity of these cells. Influenza‐specific T cells of HLA‐A2^+^ influenza A‐infected individuals were labelled with dextramers containing the immunodominant influenza A epitope GILGFVFTL (Gotch et al., 1987). Whereas these specific cells could be detected in all other T‐cell subsets, most predominantly in the T_CM_ and T_EM_ subsets (Figure 6i), we observed no GILGFVFTL‐specific cells within the KIR^+^RA^+^ T‐cell subset. These findings hint toward a potential bystander cell role for activated KIR^+^RA^+^ T cells during an event of influenza infection as they are not specific for the immunodominant GILGFVTL epitope, although it has to be noted that they may recognize minor other influenza virus‐derived epitopes.

### 2.7 KIR^+^RA^+^ T cells are highly activated in SARS‐CoV‐2 infected adults suffering from COVID‐19

Additionally, we had the unique opportunity to analyze the KIR^+^RA^+^ T‐cell subset in adults infected with SARS‐CoV‐2 (32–52 years of age, *n* = 9, Table S2) (van der Hoek et al., 2020). In these individuals, we also observed significant activation of KIR^+^RA^+^ T cells during the early phase of COVID‐19 compared to the follow‐up at 2 and 4 weeks and compared to SARS‐CoV‐2 negative controls (Figure 6j). Collectively, these findings show that activation of KIR^+^RA^+^ T cells is a major phenomenon in respiratory viral infectious diseases.

### 2.8 Activation of KIR^+^RA^+^ T cells correlates with prolonged influenza A‐induced symptoms

We reasoned that accumulation of suppressive KIR^+^RA^+^ T cells that become activated during respiratory viral infectious disease may suppress protective CD8^+^ T‐cell responses and thereby prolong virus‐induced respiratory disease in older adults. Therefore, we monitored the occurrence and duration of ILI‐associated symptoms (cough, fever, myalgia, nasal congestion, sore throat, difficulty breathing, and headache) from which we calculated an overall symptom score in the influenza A‐infected older individuals (Figure S7d). The frequency of CD69^+^ KIR^+^RA^+^ T cells during ILI positively correlated with the overall symptom score, hinting toward a link between the average duration of symptoms and the presence of activated KIR^+^RA^+^ T cells (Figure 6k). Moreover, the presence of these activated cells positively correlated with the duration of coughing (Figure 6l), which may suggest delayed viral clearance. However, the frequency of activated KIR^+^RA^+^ T cells did not correlate with the duration of fever (Figure 6m) or the other symptoms assessed separately (data not shown). In addition, the frequency of CD69^+^ T_EMRA_ cells also correlated with the duration of coughing, and a trend toward correlation with the symptom score (Figure S8) despite lower frequency of T_EMRA_ cells than KIR^+^RA^+^ T cells expressing CD69 at the time of influenza (Figure 6c). Taken together, these data exemplify potential clinical relevance of KIR^+^RA^+^ T cells in older adults suffering from a viral respiratory infection.

## 3 DISCUSSION

Here we identify a novel human CD8^+^ T‐cell subset with regulatory properties, which we refer to as KIR^+^RA^+^ T cells based on their unique expression of KIR and CD45RA. We show that KIR^+^RA^+^ T cells accumulate during aging and may contribute to diminished CD8^+^ T‐cell responses observed in older adults. Furthermore, our findings show that KIR^+^RA^+^ T cells become activated in older adults suffering from viral respiratory diseases and that this activation correlates with duration of respiratory symptoms as exemplified by influenza A infection. Together, our findings indicate that KIR^+^RA^+^ T cells are a hallmark of aging with relevance for viral respiratory infections at older age.

Human CD8^+^ CD45RA^+^ T cells that express KIR and/or NKG2A have been collectively described as one population of “virtual memory T cells” that has been reported to increase with age (Jacomet et al., 2015; Quinn et al., 2018; White et al., 2016). We now show that the virtual memory T‐cell subset comprises two distinct cell subsets: KIR^+^RA^+^ T cells that accumulate with age and NKG2A^+^RA^+^ T cells that decline with age. Our data show that KIR^+^RA^+^ T cells should be considered as a distinct and therefore unique CD8^+^ T‐cell subset. Hence, the previously reported rise of virtual memory T cells would therefore comprise the KIR^+^ cells rather than the distinct subset of NKG2A^+^ cells. Moreover, KIR^+^RA^+^ T cells constitute 30% of the T‐cell subset that has been designated T_EMRA_ cells (Hamann et al., 1997; Larbi & Fulop, 2014; Sallusto et al., 1999). Since KIR^+^RA^+^ T cells have remained hidden among the T_EMRA_ subset, our findings indicate that part of previously reported age‐related increase of the conventional T_EMRA_ subset and expression of TIGIT by these T_EMRA_ cells (Song et al., 2018) may actually be caused by the age‐related increase in KIR^+^RA^+^ T cells.

The negative correlation of KIR^+^RA^+^ T cells with CD8^+^ T‐cell proliferation led us to hypothesize that KIR^+^RA^+^ T cells may regulate CD8^+^ T‐cell proliferation. Indeed, KIR^+^RA^+^ CD8^+^ T cells appeared to express markers previously reported to be expressed by suppressive CD4^+^ and CD8^+^ T_REGS_ such as TIGIT, Helios, and CD122 (Dai et al., 2010; Fuhrman et al., 2015; Kim et al., 2015; Machicote et al., 2018; Rifa'i et al., 2004). Moreover, we show that KIR^+^RA^+^ T cells suppress proliferation of other CD8^+^ T cells, in agreement with the hypothesis of age‐related accumulation of T_REGS_, which is thought to hamper protective T‐cell responses (Gregg et al., 2005; Lages et al., 2008; Sharma et al., 2006; Simone et al., 2008). KIR^+^RA^+^ T cells have until now been regarded to be part of the virtual memory cell subset. It has been speculated that murine virtual memory cells may acquire tolerogenic mechanisms (Drobek et al., 2018), whereas another report showed that murine virtual memory cells can mediate protective immunity by killing (White et al., 2016). An explanation for these contrasting results may be the existence of different subsets among murine virtual memory T cells like the KIR^+^RA^+^ and NKG2A^+^RA^+^ subsets we report among human virtual memory T cells.

A question that remains is by what mechanism KIR^+^RA^+^ T cells suppress proliferation of other CD8^+^ T cells. First, we observed that KIR^+^RA^+^ T cells highly express the co‐inhibitory receptor TIGIT. As TIGIT expression has been reported to directly and indirectly contribute to the stability and suppressive capacity of CD4^+^ T_REGS_ (Fuhrman et al., 2015; Joller et al., 2014), these findings may indicate a similar role for TIGIT in suppressive KIR^+^RA^+^ T cells we present here. Second, Helios expression has been shown to be required for the survival and suppressive capacity of CD8^+^ T_REGS_ in mice (Kim et al., 2015). As we observed that KIR^+^RA^+^ T cells highly express Helios, targeting Helios may be an additional candidate for unraveling the suppressive mechanism of KIR^+^RA^+^ T cells. Lastly, killing of other cells by release of granzymes and perforins has been described as one of the mechanisms by which T_REGS_ may exert immune suppression (Gondek et al., 2005; Grossman et al., 2004). This mechanism may also be involved in regulation by KIR^+^RA^+^ T cells since they express CD107a, indicative of enhanced release of granules containing cytotoxic factors, and their transcriptome is enriched with transcripts for pathways involved in killing of lymphocytes.

Bystander activation is generally defined as the activation of a T cell through a mechanism which is independent of specific T‐cell receptor (TCR) stimulation. A study in mice has shown that virtual memory T cells act as bystander cells during infection (White et al., 2016). The KIR^+^RA^+^ T cells we describe here can be considered as a subset of the T cells defined as virtual memory cells. We here show that KIR^+^RA^+^ CD8^+^ T cells present in influenza A‐infected older adults are highly activated during acute infection, but not specific for the highly conserved and immunodominant influenza A epitope GILGFVFTL. This provides evidence in favor of a potential bystander role of these cells, although it is still possible that KIR+RA+ cells recognize minor other influenza‐derived epitopes that we may have missed in our analyses. Interestingly, we also found a large proportion of activated KIR^+^RA^+^ T cells in adults suffering from a primary event of COVID‐19 caused by SARS‐CoV‐2 infection. Only a minority (up to 20%) of individuals are expected to have a small fraction of cross‐reactive antigen‐specific memory CD8^+^ T cells directed against this virus, which further supports a bystander role for KIR^+^RA^+^ T cells (Grifoni et al., 2020). The exact factors inducing bystander activation remain to be elucidated, but IL‐15 has been implicated (White et al., 2016). Together, our findings suggest that the accumulation of KIR^+^RA^+^ T cells with age may play a role in a wide range of respiratory viral infections.

To understand the in vivo clinical relevance of KIR^+^RA^+^ T cells, we analyzed the presence and dynamics of activation of these cells in older adults suffering from respiratory viral infection. Our findings show that KIR^+^RA^+^ T cells become a highly activated T‐cell subset during respiratory infections caused by various viruses. It has been shown previously that coughing associates with increased viral load in the lungs (Hijano et al., 2019). We observed a positive correlation between the level of activated KIR^+^RA^+^ CD8^+ ^T cells and duration of coughing, which may suggest hampered clearance of infected lung cells. As cytotoxic CD8^+^ T cells clear virally infected cells and thereby reduce the severity of disease (Graham et al., 1991; Slutter et al., 2013; Sridhar et al., 2013; Wang et al., 2015), suppression of their responses by KIR^+^RA^+^ T cells may prolong illness. Indeed, CD4^+^ T_REGS_ have been reported to suppress CD8^+^ effector cells in influenza A‐infected mice (Brincks et al., 2013). Recently, a function of TIGIT was identified in the prevention of pathological tissue damage in an infectious disease setting (Schorer et al., 2020). This suggests that TIGIT^Hi^ KIR^+^RA^+^ T cells may also be advantageous in preventing excessive lung damage. Therefore, the role of KIR^+^RA^+^ T cells may be two‐sided: they may be needed to limit lung damage caused by cytotoxic immune cells, whereas an overrepresented activation of KIR^+^RA^+^ T cells may hamper effective clearance of pathogens. Establishing correlates of respiratory disease is highly important for developing strategies to reduce the burden of disease following respiratory infections as elderly are at higher risk of severe disease, hospitalization, and death (reviewed in McElhaney et al., 2016). Our findings indicate that KIR^+^RA^+^ T cells may serve as such a correlate of respiratory disease. The KIR^+^RA^+^ T‐cell proportion is smaller in younger individuals and symptoms are in general less severe at younger age. Although we could only analyze influenza‐like symptoms in older adults in our cohorts, it is tempting to speculate that the lower abundance of KIR^+^RA^+^ T cells at younger age may relate to lower severity of symptoms generally found at younger age. Moreover, our findings warrant future investigation on elucidating their immunoregulatory mechanisms as well as their role at the site of infection. Finally, future studies in large cohorts will further reveal how functionalities of human T cells relate to severity of infectious diseases, and how co‐morbidities and other confounders that occur at older age may interfere with this.

Our study adds a new T‐cell subset to the current panel of other aging‐related alterations of the immune system, such as the decline of the naive pool among the CD8^+^ T‐cell population that our data confirmed and has widely been considered to also contribute to reduced immunity to novel viral infections at old age. Future studies may provide insight in how newly identified T‐cell subsets such as KIR^+^RA^+^ T cells may interact with different other T‐cell subsets, B cells producing antibodies, and innate immune functions. Understanding of the impact of aging on all these subsets and their complex interactions may provide novel directions toward reducing risk of severe infectious disease at old age.

In summary, our findings support a model for declining CD8^+^ T‐cell responses with age where accumulation of regulatory KIR^+^RA^+^ T cells may in part determine the outcome of protective T‐cell responses against respiratory infection. These cells may therefore serve as target for new preventive or therapeutic strategies.

## 4 EXPERIMENTAL PROCEDURES

### 4.1 Study design

#### 4.1.1 Older adults with Influenza‐like illness and matched controls

Part of the samples investigated in the current study were embedded in a trial that monitored Influenza‐like illness (ILI) in community‐dwelling older adults (ILI cohort; Netherlands Trial Register NL4666) (van Beek et al., 2017). The study was performed according to Good Clinical Practice, the Declaration of Helsinki. The study was approved by the ethical committee METC Noord‐Holland and written informed consent was obtained from all participants. There were no exclusion criteria for this study. Participants were 60 years and older, and were instructed to report ILI‐associated symptoms according to the Dutch Pel criteria (Pel, 1965) (fever ≥37.8°C with at least coughing, myalgia, nasal congestion, sore throat, difficulty breathing, or headache) as soon as possible after the symptoms started. Blood samples were drawn within 72 hr after the symptom report (acute phase of infection), as well as approximately 2 and 8 weeks after the initial report. The presence or absence of ILI‐associated symptoms was monitored during each visit. Nasopharyngeal and oropharyngeal swabs were taken at all three visits to identify the pathogen causing the ILI symptoms by qPCR as described below. Symptomatic participants reported on in this study are a subset of the ILI cohort. The selection was based on the presence of the type of respiratory virus as a single infection at the acute phase of infection and absence of all ILI‐causing pathogens at 2 and 8 weeks after the initial report. Respiratory viruses included in our selection were real‐time PCR‐based Multiplex Ligation‐dependent Probe Amplification (MPLA)‐confirmed cases of Influenza A virus (*n* = 15), Influenza B virus (*n* = 5), Respiratory Syncytial Virus (*n* = 2), Human Metapneumovirus (hMPV) (*n* = 4), seasonal Coronavirus (*n* = 5), or Rhinovirus (*n* = 5) infection (RespiFinder® Smart 22 kit, Pathofinder) (Table S1). Of these participants, the self‐reported occurrence and duration of ILI‐associated symptoms were used to calculate an overall symptom score by totaling the duration of all symptoms per individual divided by the number of symptoms of the individual. Participants who were asymptomatic and laboratory‐confirmed respiratory infection negative were sampled and used as healthy controls in this ILI cohort.

#### 4.1.2 Healthy individuals

Blood samples obtained from healthy (asymptomatic) individuals (*n* = 50, 21–82 years) investigated in the current study were either derived from the aforementioned ILI cohort, or from healthy blood donors (Sanquin Blood Supply Foundation) or from healthy participants of a trial performed at the RIVM (Netherlands Trial Register NL1952) (Rosendahl Huber et al., 2018). Overall, the samples investigated in this study were relatively evenly distributed over age and sex (Table S3). All healthy and symptomatic participants from the aforementioned cohorts that were included in this study were tested for cytomegalovirus (CMV) antibodies by an in‐house Multiplex Immunoassay (Tcherniaeva et al., 2018). Only participants who tested negative were included in the study, to prevent bias toward CMV‐induced T‐cell alterations.

#### 4.1.3 SARS‐CoV‐2 infected adults with COVID‐19

With the recent COVID‐19 pandemic caused by the novel SARS‐CoV‐2, we expanded our analyses of KIR^+^RA^+^ T cells by investigating the presence and phenotype of these cells in the blood of adults with COVID‐19. Whole blood of PCR‐confirmed SARS‐CoV‐2 infected participants (32–52 years of age, *n* = 9) and that of asymptomatic household members who tested negative for SARS‐CoV2 (indicated as healthy controls) (16–51 years of age, *n* = 9) was obtained as part of the First Few X (FFX) cases of SARS‐CoV‐2 infection in the Netherlands (Table S2) (van der Hoek et al., 2020). Blood samples were drawn within the first two weeks after COVID‐19‐like symptoms were reported (acute phase of infection), as well as two and four weeks thereafter. The study was approved by the Utrecht ethical committee (METC Utrecht; NL13529.041.06).

### 4.2 PBMC isolation and flow cytometry

Peripheral blood mononuclear cells were isolated by density gradient (Ficoll‐Hypaque, Amersham Biosciences) from heparinized blood or buffy coats and stored at −135°C in 10% dimethyl sulfoxide (DMSO, Sigma‐Aldrich) and 10% fetal calf serum (FCS) until further use. For ex vivo flow cytometric analyses, frozen PBMCs were thawed at 37°C and were transferred to RPMI‐1640 medium (GIBCO, Thermo Fisher Scientific) supplemented with 10% FCS and Penicillin‐Streptomycin‐Glutamine (P/S/G) and washed twice. Thawed cells were then rested in medium for 30 min at room temperature after which the number of viable cells was determined on a Coulter Counter (Beckman). For ex vivo analyses on all healthy individuals, 4 × 10^5^ PBMCs of each individual were labeled for surface markers at 4°C for 30 min with saturating concentrations of antibodies targeting: CD3, CD4, CD8, CD27, CD45RA, CD122, CD226, KIR2D, KIR3DL1, NKG2A, and TIGIT (listed in Table S4) in FACS buffer (1x PBS + 0.5% BSA + 2 mM EDTA). A Fixable Viability Stain 780 (BD Horizon) was added to the labeling to identify viable cells. Cells were then fixed and permeabilized with buffers for subsequent intracellular labeling (eBioscience) at 4°C for 30 min with an antibody targeting Helios (Table S4). For ex vivo analyses on symptomatic individuals and their asymptomatic/healthy controls, 2–6 × 10^6^ PBMCs were first labeled with an HLA‐A2 GILG‐dextramer (A*0201/GILGFVFTL dextramer, FITC‐conjugated, Immudex) for 20 min at room temperature. Cells were then labeled for surface markers at 4°C for 30 min with saturating concentrations of antibodies targeting: CD3, CD4, CD8, CD27, CD45RA, CD69, KIR2D, KIR3DL1, NKG2A, and TIGIT in FACS buffer, before intracellular labeling of Helios as described above (listed in Table S4). A Fixable Viability Stain 780 (BD Horizon) was used to identify viable cells. Samples were measured on an LSRFortessa™ X‐20 (BD Biosciences) and data were analyzed using FlowJo software (v10.6.1, TreeStar).

### 4.3 T‐cell culture assays

#### 4.3.1 Cell sorting for T‐cell cultures

For all culture assays, we used PBMC that were collected from healthy blood donors (Sanquin Blood Supply Foundation) and thawed upon storage at −135°C. The number of viable cells in thawed PBMC samples was determined manually using trypan blue staining and Bürker‐Türk. For T‐cell suppression and T‐cell proliferation/activation assays, (part of) the cells were washed with 1xPBS and labelled with 0.5 µM CellTrace^TM^ Violet (Invitrogen) in 1x PBS per milliliter of cell suspension (10^6^ cells/ml) for 20 min at 37°C to track their proliferation. Ice‐cold RPMI‐1640 medium (+10% FCS, +P/S/G) was added, and cells were rested at room temperature for 5 min. Cells were centrifuged at 400 *g* for 5 min and washed with RPMI‐1640 medium (+10% FCS, +P/S/G) three times. Cells were then labeled for surface markers at 4°C for 30 min with saturating concentrations of antibodies targeting: CD4, CD8, CD45RA, CD56, KIR2D, KIR3DL1, and NKG2A (listed in Table S4), before suspension in FACS buffer + 25 mM HEPES. Cell sorting was performed using the FACSMelody™ (BD Biosciences). CD4^−^CD56^−^CD8^+^ single cells were sorted into the following subsets: KIR^+^RA^+^ T cells (CD8^+^CD45RA^+^KIR^+^NKG2A^−^), NKG2A^+^RA^+^ T cells (CD8^+^CD45RA^+^KIR^−^NKG2A^+^), and KIR^−^NKG2A^−^ T cells (CD8^+^KIR^−^NKG2A^−^).

#### 4.3.2 T‐cell stimulation: proliferation and activation assays

Total CellTrace™ Violet (Invitrogen) labeled PBMCs were cultured in the presence of 0.005 µg/ml plate‐bound purified mouse anti‐human CD3 (Clone HIT3α, BD Biosciences) in RPMI‐1640 medium in U‐bottom plates (2 × 10^5^ cells/well). For analyses of responsiveness of KIR^+^RA^+^ CD8^+^ T cells to cytokines, PBMC were cultured without anti‐human CD3, but in the presence of 100 ng/ml recombinant human interleukin‐2 (Miltenyi Biotec), or 10 ng/ml recombinant human interleukin‐15 (R&D Systems) or culture supernatant derived from PBMC (2 × 10^5^/well) that had been stimulated with anti‐CD3/CD28‐coupled Dynabeads™ (1 bead: 12 cells) for 3 days. FACS‐sorted and CellTrace™ Violet‐labeled KIR^+^RA^+^, NKG2A^+^RA^+^ and KIR^−^NKG2A^−^ T‐cell subsets were cultured in the presence of anti‐CD3/CD28‐coupled beads (Dynabeads™, Gibco) at a 1:12 bead/cell ratio in RPMI‐1640 medium in U‐bottom plates for 3 days. After 3 days of culturing, cells were first labeled for surface markers at 4°C for 30 min with saturating concentrations of a combination of antibodies targeting: CD4, CD8, CD45RA, CD69, KIR2D, KIR3DL1, NKG2A, and TIGIT in FACS buffer, before intracellular labeling of CD3 and Helios (listed in Table S4). A Fixable Viability Stain 780 (BD Horizon) was used to identify viable cells. Samples were measured on an LSRFortessa™ X‐20 (BD Biosciences) and data were analyzed using FlowJo software (v10.6.1, TreeStar).

#### 4.3.3 T‐cell suppression assays

Suppression assays were performed by culturing FACS‐sorted and CellTrace™ Violet‐labeled KIR^−^NKG2A^−^ CD8^+^ T cells (responder cells) with KIR^+^NKG2A^−^ CD8^+^ T cells (suppressor cells) that were either CellTrace™ Violet‐labeled or not. Cells were cultured together in a range of suppressor‐to‐responder ratios from 1:1 to 1:16 in a total amount of 10,000 cells/well. During culture, cells were stimulated with anti‐CD3/CD28‐coupled beads (Dynabeads™, Gibco) at a 1:12 bead/cell ratio in U‐bottom plates. After 3 days of culturing, cells were labeled for surface markers at 4°C for 30 min with saturating concentrations of antibodies targeting: CD3, CD8, CD45RA, CD69, KIR2D, KIR3DL1, NKG2A, and TIGIT in FACS buffer (listed in Table S4). A Fixable Viability Stain 780 (BD Horizon) was used to identify viable cells. Samples were measured on an LSRFortessa™ X‐20 (BD Biosciences) and acquired flow cytometry data were analyzed using FlowJo software (TreeStar). Suppression of responder proliferation by suppressor cells was calculated as follows: ((%proliferation responder only – % proliferation with suppressor cells)/(%proliferation responder only)) × 100%.

### 4.4 Intracellular cytokine assay

Peripheral blood mononuclear cells were added to U‐bottom plates (1 × 10^6^ cells/well) in RPMI‐1640 medium in the presence of anti‐CD107a labeling (Biolegend), and exposed to phorbol‐12‐myristate‐13‐acetate (PMA, 25 ng/ml, Sigma‐Aldrich) with ionomycin (250 ng/ml, Sigma‐Aldrich) for 6 hr at 37°C or to medium only as control. After 2 hr of incubation, Brefeldin A (1:100, Sigma‐Aldrich) and Monensin (1:150, GolgiStop, BD Biosciences) were added and present during the final 4‐hr incubation period. After incubation, cells were labeled for surface makers for 30 min at 4°C with saturating concentrations of antibodies targeting: CD8, CD27, CD45RA, KIR2D, KIR3DL1, and NKG2A in FACS buffer, followed by intracellular labeling of CD3 and the cytokines IL‐2, IFN‐γ, and TNF‐α (listed in Table S4). A Fixable Viability Stain 780 (BD Horizon) was used to identify viable cells. Samples were measured on an LSRFortessa™ X‐20 (BD Biosciences) and data were analyzed using FlowJo software (v10.6.1, TreeStar).

### 4.5 RNA sequencing

#### 4.5.1 Cell sorting for RNA sequencing

The number of viable cells in thawed PBMC samples was determined manually using trypan blue staining and Bürker‐Türk as described above. Cells were labeled for surface markers at 4°C for 30 min with saturating concentrations of antibodies targeting: CD3, CD4, CD8, CD19, CD27, CD45RA, CD56, KIR2D, KIR3DL1, and NKG2A in FACS buffer (listed in Table S4). Cells were suspended in FACS buffer +25 mM HEPES. First, viable single CD8^+^ T cells were selected as CD56^−^CD4^−^CD19^−^CD3^+^CD8^+^ cells. These CD8^+^ T cells were then subdivided into T_NAIVE_ cells (CD8^+^CD45RA^+^KIR^−^NKG2A^−^CD27^+^), T_EMRA_ cells (CD8^+^CD45RA^+^KIR^−^NKG2A^−^CD27^−^), KIR^+^RA^+^ T cells (CD8^+^CD45RA^+^KIR^+^NKG2A^−^), and NKG2A^+^RA^+^ T cells (CD8^+^CD45RA^+^KIR^−^NKG2A^+^), which were sorted separately into tubes containing RPMI‐1640 medium (+20% FCS, +P/S/G). Cell sorting was performed using the FACSMelody™ (BD Biosciences).

#### 4.5.2 Library preparation

FACS‐sorted T_NAIVE_, T_EMRA_, KIR^+^RA^+^, and NKG2A^+^RA^+^ T‐cell subsets of six healthy donors (ages: 30, 57, 61, 63, 65, 66 years) were centrifuged at 485 *g* for 15 min and lysed in buffer RLT (Qiagen) containing 1% β‐mercaptoethanol. Total RNA was extracted from the cell lysates by using the RNeasy kit (Qiagen) according to the manufacturer’s protocol. RNA integrity was assessed by using the RNA 6000 Pico kit (Agilent Technologies) on a 2100 BioAnalyzer (Agilent Technologies). All RNA Integrity Number (RIN) scores were >7.0. cDNA synthesis and amplification were performed by using the SMART‐Seq® v4 Ultra® Low Input RNA kit for sequencing (Takara) and AMPure XP beads (Beckman Coulter) were used to purify the samples. Proper size distribution of the fragments acquired was verified using the High Sensitivity DNA kit (Agilent Technologies). Next, 1 ng of cDNA was used to prepare a Nextera XT DNA library according to the manufacturer’s instruction (Illumina). Libraries were subsequently validated for fragment size using QIAxcel DNA Screening Kit (Qiagen) and quantified using RT‐qPCR with a KAPA Library Quantification kit (KK4824, Roche/KAPA Biosystems). 23 libraries were pooled at equimolar concentrations and sequenced using the Illumina NextSeq 500/550 High Output Kit v2.5 (single‐end, 75‐cycles). Basecalling and demultiplexing were performed using bcl2fastq2 Conversion Software v2.20, and demultiplexed FASTQ files which were generated based on sample‐specific barcodes (>14 million reads/sample).

#### 4.5.3 RNA sequencing analysis

We used an in‐house pipeline to analyze the RNA sequencing data. First, reads were mapped to the human reference genome (GRCh38, release 12) using STAR (version 2.6.0) (Dobin et al., 2013). The number of mapped reads were counted for each gene and compiled into an expression matrix using featureCounts (version 1.6.1) (Liao et al., 2014). The count table was used for statistical analysis and identification of DEG using DESeq2 (v1.1) (Love et al., 2014). Genes were considered significantly different with a false discovery rate (FDR) <0.1. The RNA‐seq data discussed in this article will be deposited and made accessible in the National Center for Biotechnical Information Gene Expression Omnibus 23 upon acceptation for publication. Ingenuity Pathway Analysis (IPA, Qiagen) was used to investigate the relationships between selected pairs of T‐cell subsets. Gene expression profiles of T_EMRA_, KIR^+^RA^+^, and NKG2A^+^RA^+^ T‐cell subsets were first separately compared to the gene expression profile of T_NAIVE_ cells. These comparisons were then aligned in a heat map to identify shared and unique molecular and cellular functions of T_EMRA_, KIR^+^RA^+^, and NKG2A^+^RA^+^ T cells. For this enrichment analysis, an absolute *z*‐score of >2.0, a False Discovery Rate (FDR) of 0.10, and a ‐Log(*p*‐value) of >1.5 was used. *p*‐values were calculated by Fisher’s Exact Test.

### 4.6 Whole blood flow cytometry in COVID‐19 FFX study

Whole blood of participants in the FFX study investigating the pandemic of SARS‐CoV‐2 in the Netherlands was obtained from adults suffering from COVID‐19 with a PCR‐confirmed SARS‐CoV‐2 infection and that of asymptomatic SARS‐CoV‐2‐PCR‐negative household members. 100 µl whole blood of each donor was labeled for surface markers at room temperature for 15 min with saturating concentrations of antibodies targeting: CD3, CD4, CD8, CD27, CD45, CD45RA, CD69, KIR2D, KIR3DL1, and NKG2A (listed in Table S4) in Brilliant Stain Buffer (BD Biosciences). Erythrocytes were lysed with 1 ml lysing solution (BD Biosciences) for 15 min at room temperature. To fasten measurement of stained PBMC, cell suspensions were concentrated by centrifugation at 400 *g* for 5 min and resuspension in the 400 µl remaining after removal of 700 µl of fluid phase after centrifugation. Samples were measured on an LSRFortessa™ X‐20 (BD Biosciences) and data were analyzed using FlowJo software (v10.6.1, TreeStar).

### 4.7 Statistics

Statistical analysis was performed using GraphPad Prism version 8.4.1. The appropriate parametric or non‐parametric tests were used based on the tested normality of distribution of the data. Two groups were compared by using Mann–Whitney *U* test (unpaired), or Wilcoxon Test (paired) with two‐sided *p*‐values. When comparing more than two groups, paired analyses were performed with either row‐matched one‐way ANOVA with Geisser‐Greenhouse correction and Dunnett’s post‐test, or with Friedman Test with Dunn’s post‐test. Correlations between variables were analyzed using Spearman’s rank correlation coefficient (*r*). Linear regression analysis was performed to generate lines of best fit. Statistical significance was considered when *p* < 0.05, with *p < 0.05, **p < 0.01, ***p < 0.001, and ****p < 0.0001. All data presented in bar graphs are depicted as mean ± *SD*.

## CONFLICT OF INTEREST

The authors declare no conflict of interest.

## AUTHOR CONTRIBUTIONS

DKJP, NAMS, and TG designed the study, DKJP, NAMS, JH, VK, RJP, RM, and TG performed the experiments, JvB designed and facilitated the sample collection of parental clinical cohort studies, DKJP, NAMS, RM, and TG analyzed the data, DKJP, NAMS, RM, JvB, DvB, JdW, and TG interpreted the data, DKJP and TG wrote the manuscript, DKJP, RM, JvB, DvB, JdW, and TG reviewed and edited the manuscript.

## Supporting information

Supplementary Material

## Data Availability

The data presented in this study are available from the corresponding author on reasonable request. RNA‐seq data have been deposited and made accessible in the National Center for Biotechnical Information Gene Expression Omnibus (GEO) by series record accession number GSE174779. For information on GEO linking and citing, please refer to: https://www.ncbi.nlm.nih.gov/geo/info/linking.html.
