# Supplementary material for: Regulatory KIR+RA+ T cells accumulate with age and are highly activated during viral respiratory disease
Source: Aging Cell. 2021 May 27;20(6):e13372. doi: 10.1111/acel.13372 (PMC8208794; doi:10.1111/acel.13372)
Supplement: Supplementary file 1 — Supplementary Material [file ACEL-20-e13372-s001.docx]

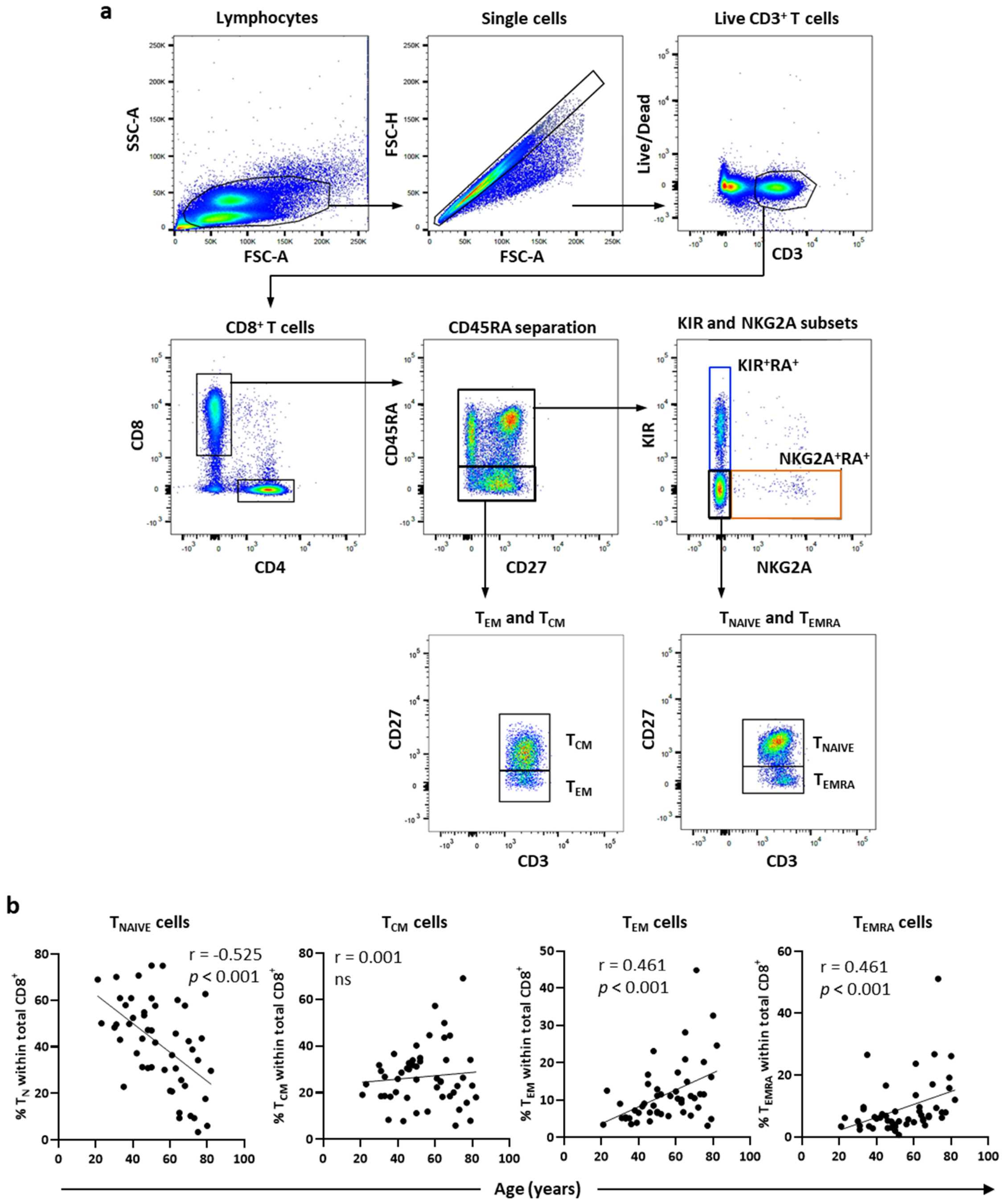

Supplementary Figure 1. Gating strategy of CD8$^+$ T-cell subsets and their proportion with age

(**a**) Gating strategy towards the six CD8$^+$ T-cell subsets in healthy individuals (n=50, 20-82 years of age) using flow cytometry. CD8$^+$ T cells were identified based on gating of total lymphocytes, single cells, and live CD3$^+$ T cells. CD8$^+$ T cells were then separated into CD45RA$^+$ and CD45RA$^-$ cells. Within the CD45RA$^+$ T-cell population, KIR$^+$RA$^+$ T cells (CD3$^+$CD8$^+$CD45RA$^+$KIR$^+$NKG2A$^-$, blue gate) and NKG2A$^+$RA$^+$ T cells (CD3$^+$CD8$^+$CD45RA$^+$KIR$^-$NKG2A$^+$, orange gate) were identified. Remaining CD45RA$^+$ KIR$^-$ NKG2A$^-$ cells were divided into naive T cells (T_{NAIVE}: CD3$^+$CD8$^+$CD45RA$^+$KIR$^-$NKG2A$^-$CD27$^+$) and terminally differentiated effector memory cells re-expressing RA (T_{EMRA}: CD3$^+$CD8$^+$CD45RA$^+$KIR$^-$NKG2A$^-$CD27$^-$). CD45RA$^-$ CD8$^+$ T cells were separated into central memory cells (T_{CM}: CD3$^+$CD8$^+$CD45RA$^-$CD27$^+$) and effector memory cells (T_{EM}: CD3$^+$CD8$^+$CD45RA$^-$CD27$^-$). (**b**) Relationship between the frequency of T_{NAIVE}, T_{CM}, T_{EM}, or T_{EMRA} cells within the total CD8$^+$ T-cell population and age. Correlations (*r* values) were assessed by Spearman test.

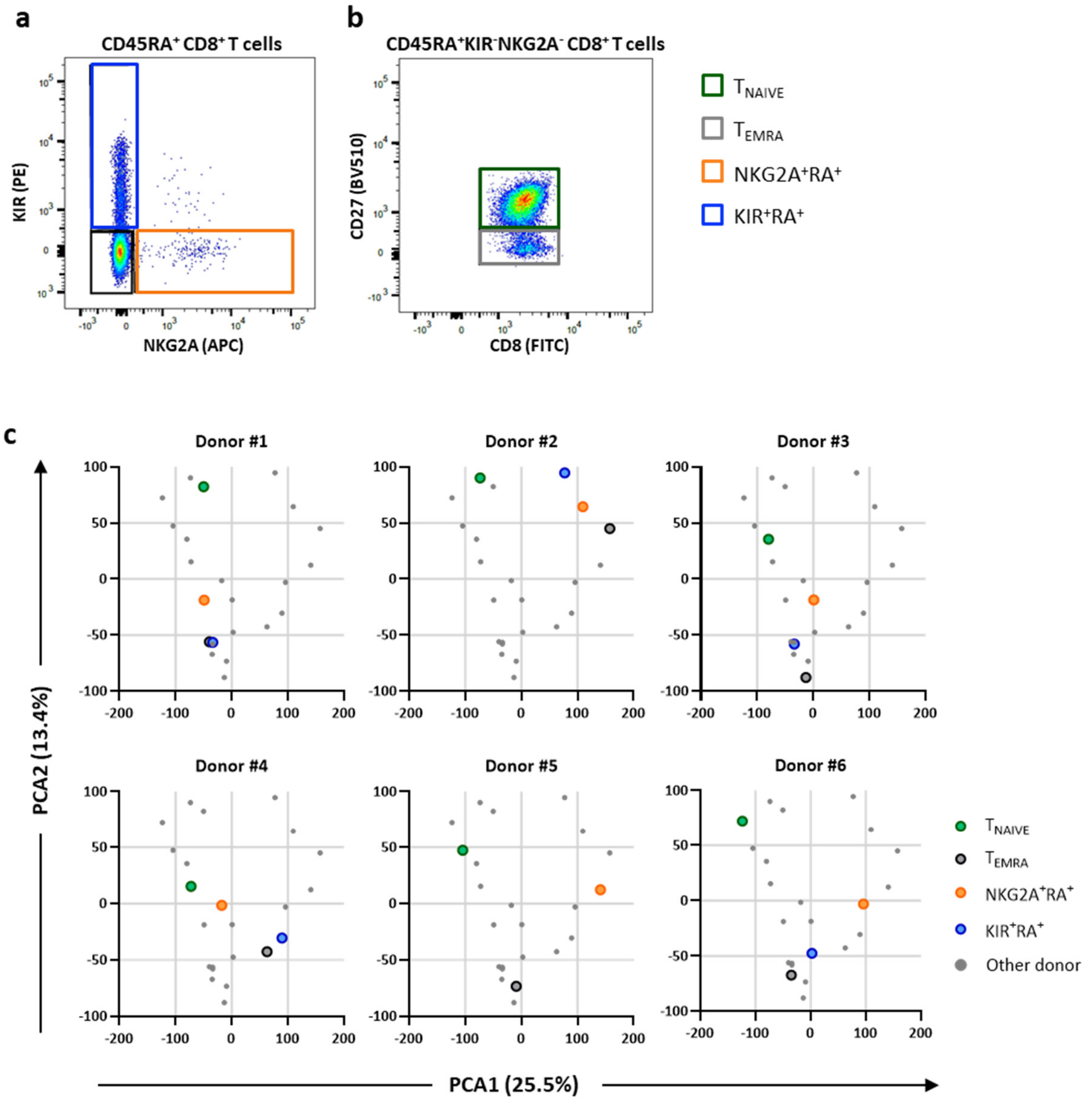

Supplementary Figure 2. Sorted T-cell subsets and donor variation observed in unsupervised principal component analysis (PCA).

(**a,b**) Flow cytometry plots indicate the four FACS-sorted subsets, first gated on $CD56^-CD4^-CD19^-CD3^+CD8^+$ T cells: T_{NAIVE} cells ($CD8^+CD45RA^+KIR^-NKG2A^-CD27^+$), T_{EMRA} cells ($CD8^+CD45RA^+KIR^-NKG2A^-CD27^-$), KIR^+RA^+ T cells ($CD8^+CD45RA^+KIR^+NKG2A^-$), and $NKG2A^+RA^+$ T cells ($CD8^+CD45RA^+KIR^-NKG2A^+$). (**C**) Unsupervised principal component analysis (PCA) performed on the total transcriptome (34,745 genes) within each of the sorted cell subsets is shown for each of the six donors.

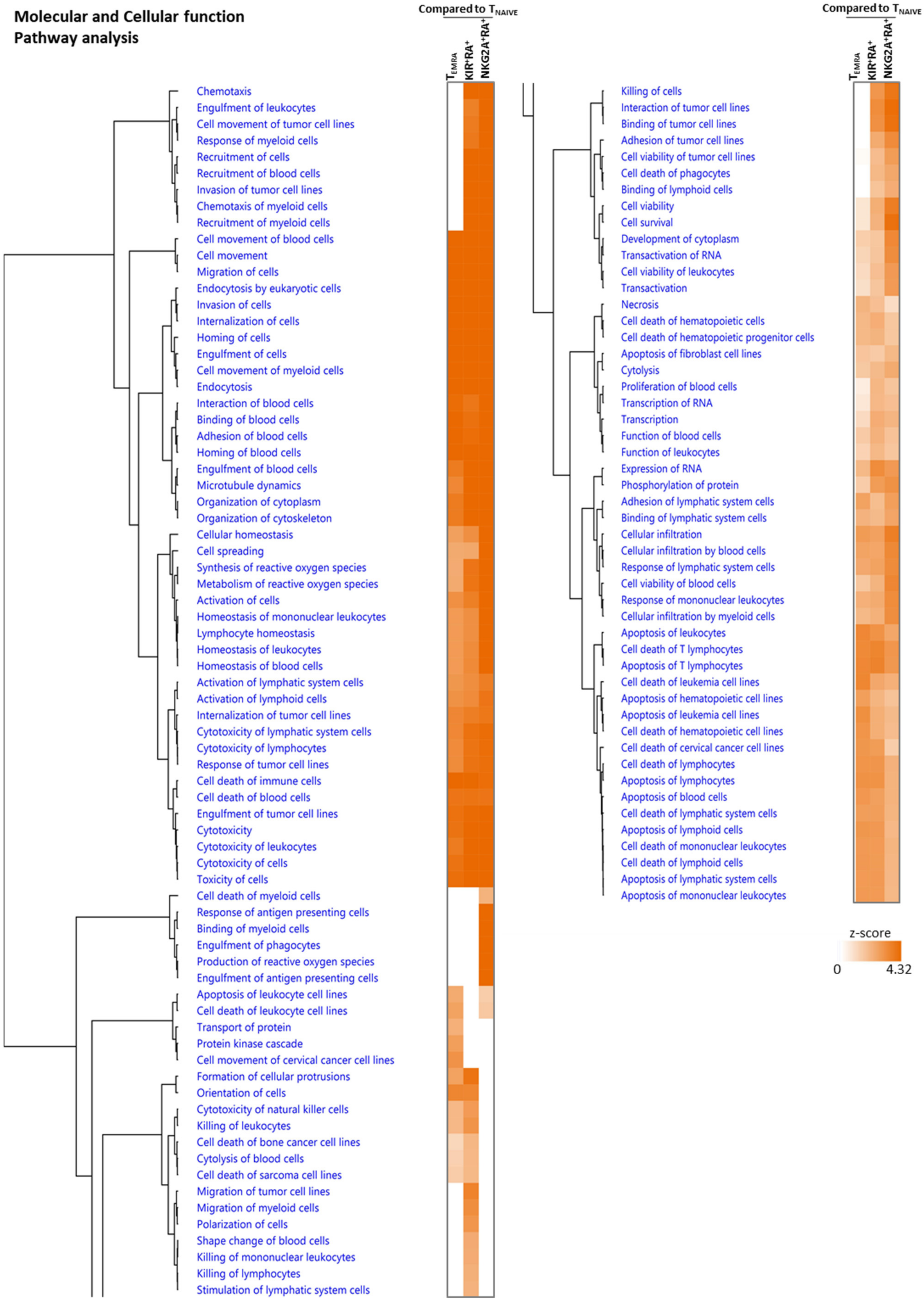
Molecular and Cellular function
Pathway analysis
Compared to T NAIVE
T EMRA
KIR+RA+
NKG2A+RA+
Chemotaxis
Engulfment of leukocytes
Cell movement of tumor cell lines
Response of myeloid cells
Recruitment of cells
Recruitment of blood cells
Invasion of tumor cell lines
Chemotaxis of myeloid cells
Recruitment of myeloid cells
Cell movement of blood cells
Cell movement
Migration of cells
Endocytosis by eukaryotic cells
Invasion of cells
Internalization of cells
Homing of cells
Engulfment of cells
Cell movement of myeloid cells
Endocytosis
Interaction of blood cells
Binding of blood cells
Adhesion of blood cells
Homing of blood cells
Engulfment of blood cells
Microtubule dynamics
Organization of cytoplasm
Organization of cytoskeleton
Cellular homeostasis
Cell spreading
Synthesis of reactive oxygen species
Metabolism of reactive oxygen species
Activation of cells
Homeostasis of mononuclear leukocytes
Lymphocyte homeostasis
Homeostasis of leukocytes
Homeostasis of blood cells
Activation of lymphatic system cells
Activation of lymphoid cells
Internalization of tumor cell lines
Cytotoxicity of lymphatic system cells
Cytotoxicity of lymphocytes
Response of tumor cell lines
Cell death of immune cells
Cell death of blood cells
Engulfment of tumor cell lines
Cytotoxicity
Cytotoxicity of leukocytes
Cytotoxicity of cells
Toxicity of cells
Cell death of myeloid cells
Response of antigen presenting cells
Binding of myeloid cells
Engulfment of phagocytes
Production of reactive oxygen species
Engulfment of antigen presenting cells
Apoptosis of leukocyte cell lines
Cell death of leukocyte cell lines
Transport of protein
Protein kinase cascade
Cell movement of cervical cancer cell lines
Formation of cellular protrusions
Orientation of cells
Cytotoxicity of natural killer cells
Killing of leukocytes
Cell death of bone cancer cell lines
Cytolysis of blood cells
Cell death of sarcoma cell lines
Migration of tumor cell lines
Migration of myeloid cells
Polarization of cells
Shape change of blood cells
Killing of mononuclear leukocytes
Killing of lymphocytes
Stimulation of lymphatic system cells
Compared to T NAIVE
T EMRA
KIR+RA+
NKG2A+RA+
Killing of cells
Interaction of tumor cell lines
Binding of tumor cell lines
Adhesion of tumor cell lines
Cell viability of tumor cell lines
Cell death of phagocytes
Binding of lymphoid cells
Cell viability
Cell survival
Development of cytoplasm
Transactivation of RNA
Cell viability of leukocytes
Transactivation
Necrosis
Cell death of hematopoietic cells
Cell death of hematopoietic progenitor cells
Apoptosis of fibroblast cell lines
Cytolysis
Proliferation of blood cells
Transcription of RNA
Transcription
Function of blood cells
Function of leukocytes
Expression of RNA
Phosphorylation of protein
Adhesion of lymphatic system cells
Binding of lymphatic system cells
Cellular infiltration
Cellular infiltration by blood cells
Response of lymphatic system cells
Cell viability of blood cells
Response of mononuclear leukocytes
Cellular infiltration by myeloid cells
Apoptosis of leukocytes
Cell death of T lymphocytes
Apoptosis of T lymphocytes
Cell death of leukemia cell lines
Apoptosis of hematopoietic cell lines
Apoptosis of leukemia cell lines
Cell death of hematopoietic cell lines
Cell death of cervical cancer cell lines
Cell death of lymphocytes
Apoptosis of lymphocytes
Apoptosis of blood cells
Cell death of lymphatic system cells
Apoptosis of lymphoid cells
Cell death of mononuclear leukocytes
Cell death of lymphoid cells
Apoptosis of lymphatic system cells
Apoptosis of mononuclear leukocytes
z-score
0
4.32

Supplementary Figure 3. Ingenuity Pathway Analysis (IPA) performed on T_{EMRA}, KIR^+RA^+ and $NKG2A^+RA^+$ T cells compared to T_{NAIVE} cells to identify molecular and cellular function pathways.

Ingenuity Pathway Analysis (IPA) was performed by comparing the differentially expressed genes (DEGs) of T_{EMRA}, KIR^+RA^+, and $NKG2A^+RA^+$ cell subsets to T_{NAIVE} cells to identify molecular and cellular functions of these cell subsets. Pathways identified in this analysis were then compared between T_{EMRA}, KIR^+RA^+, and $NKG2A^+RA^+$ T-cell subsets by aligning their hierarchical clustered heat maps to identify in which shared and unique pathways these subsets differed from each other and from T_{NAIVE} cells. Colors in the heat map indicate the z-score calculated for each identified pathway, with cutoff values: -Log(*p*-value) >1.5, z-score with an absolute value > 2.0. Statistical significance ($p < 0.05$) was calculated by Fisher's Exact Test.

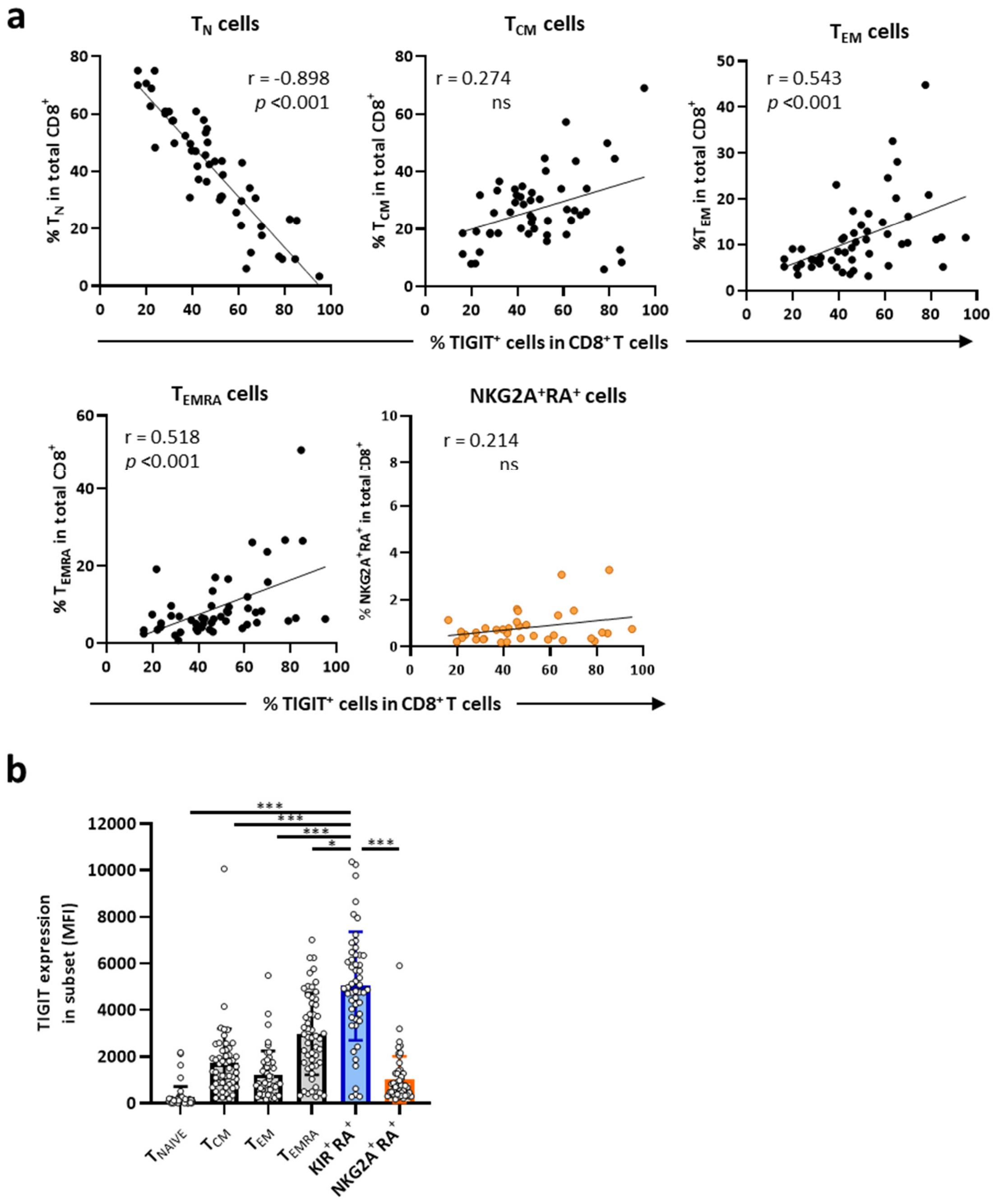

Supplementary Figure 4. Proportion of TIGIT+ cells within CD8+ T-cell subsets.

(**a**) Relationship between the proportion of T_{NAIVE}, T_{CM}, T_{EM}, T_{EMRA}, or NKG2A$^+$RA$^+$ cells within the CD8$^+$ T-cell population and the proportion of TIGIT$^+$ cells within the CD8$^+$ T-cell population. (**b**) Expression (Median Fluorescent Intensity, MFI) of TIGIT within the indicated CD8$^+$ T-cell subsets in healthy individuals (20-82 years of age, n=50). Correlations (*r* values) were assessed by Spearman test. Statistical significance of data presented in the bar graph (means ± s.d.) was determined using Friedman test (with Dunn's post-test). (**p* <0.05, ****p* <0.001).

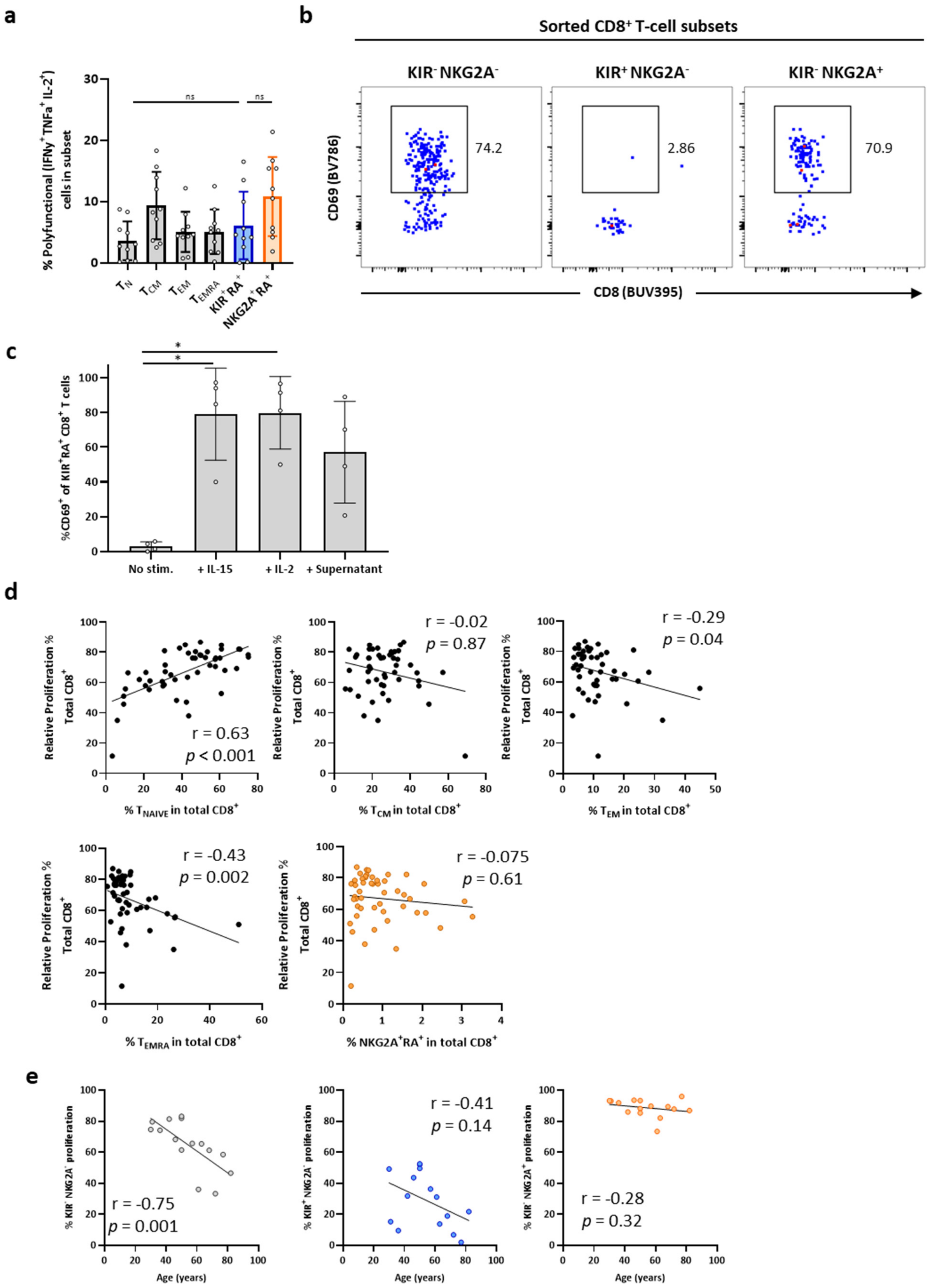

a
% Polyfunctional (IFNγ+TNFa+ IL-2+) cells in subset
ns
ns
TN
TCM
TEM
TEMRA
KIR+RA+
NKG2A+RA+
b
Sorted CD8+ T-cell subsets
KIR- NKG2A-
KIR+ NKG2A-
KIR- NKG2A+
74.2
2.86
70.9
CD69 (BV786)
CD8 (BUV395)
c
%CD69+ of KIR+RA+ CD8+ T cells
No stim.
+ IL-15
+ IL-2
+ Supernatant
d
Relative Proliferation % Total CD8+
r = 0.63
p < 0.001
% TNAIVE in total CD8+
r = -0.02
p = 0.87
% TCM in total CD8+
r = -0.29
p = 0.04
% TEM in total CD8+
r = -0.43
p = 0.002
% TEMRA in total CD8+
r = -0.075
p = 0.61
% NKG2A+RA+ in total CD8+
e
% KIR- NKG2A- proliferation
r = -0.75
p = 0.001
% KIR+ NKG2A- proliferation
r = -0.41
p = 0.14
% KIR- NKG2A+ proliferation
r = -0.28
p = 0.32
Age (years)

Supplementary Figure 5. Analysis of the KIR$^+$RA$^+$ T-cell response.

Accumulation of intracellular cytokines was measured after exposure of total PBMCs of healthy individuals (n=10) to PMA/Ionomycin for six hours. (**a**) Frequency of polyfunctional (IFN-γ$^+$ IL-2$^+$ TNF-α$^+$) cells within the indicated CD8$^+$ T-cell subsets (relative to medium control). (**b**) Frequency of CD69$^+$ cells in sorted CD8$^+$KIR$^-$ NKG2A$^-$ T cells, CD8$^+$CD45RA$^+$KIR$^+$NKG2A$^+$ T cells, and CD8$^+$CD45RA$^+$KIR$^-$NKG2A$^+$ T cells after culturing with anti-CD3/anti-CD28 beads (1:12 bead-to-cell ratio) for three days (representing findings for n=4). (**c**) Frequency of CD69$^+$ cells of KIR$^+$RA$^+$ CD8$^+$ T cells after exposure to interleukin-2, interleukin-15, or soluble factors secreted by PBMCs in response to CD3/CD28-coupled beads (n=4). (**d**) Relationships between the frequency of proliferated CD8$^+$ T cells (relative to medium control) and *ex vivo* frequency of T_{NAIVE}, T_{CM}, T_{EM}, T_{EMRA}, or NKG2A$^+$RA$^+$ cells within the total CD8$^+$ T-cell population of healthy individuals (20-82 years of age, n=50). (**e**) Relationships between the frequency of proliferated KIR$^-$ NKG2A$^-$, KIR$^+$ NKG2A$^-$, and KIR$^-$ NKG2A$^+$ CD8$^+$ T cells and age (n=16). Correlations (*r* values) were assessed by Spearman test. Statistical significance of data presented in the bar graph (means ± s.d.) was determined using row-matched one-way ANOVA (with Geisser-Greenhouse correction and Dunnett's post-test). (ns=not significant).

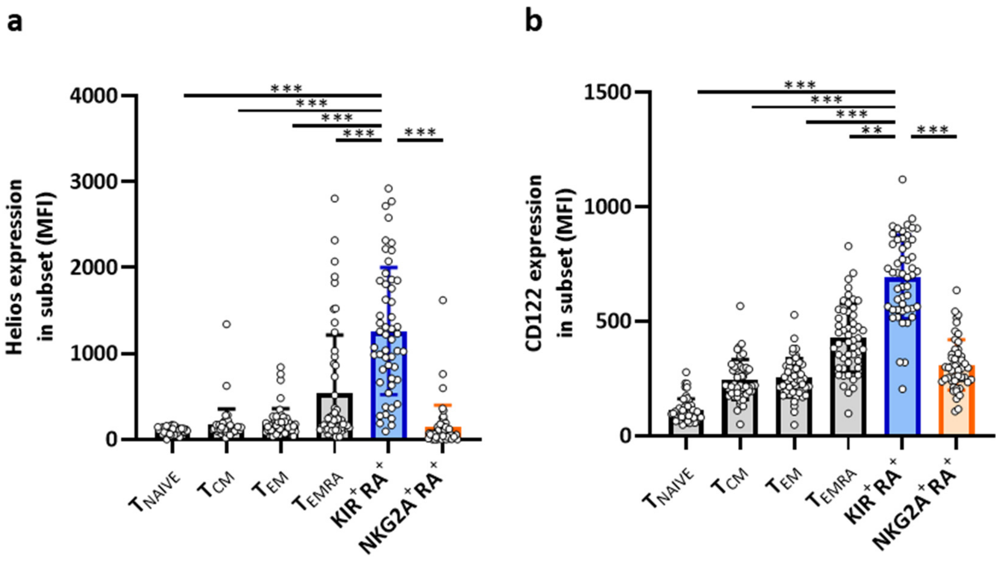

Supplementary Figure 6. KIR⁺RA⁺ T cells highly express Helios and CD122.

Expression (MFI) of (**a**) Helios and (**b**) CD122 within each of the indicated CD8⁺ T-cell subsets in healthy individuals (20-82 years of age, n=50). Statistical significance of data presented in bar graphs (means ± s.d.) was determined using Friedman test (with Dunn's post-test). (**p <0.01, ***p <0.001).

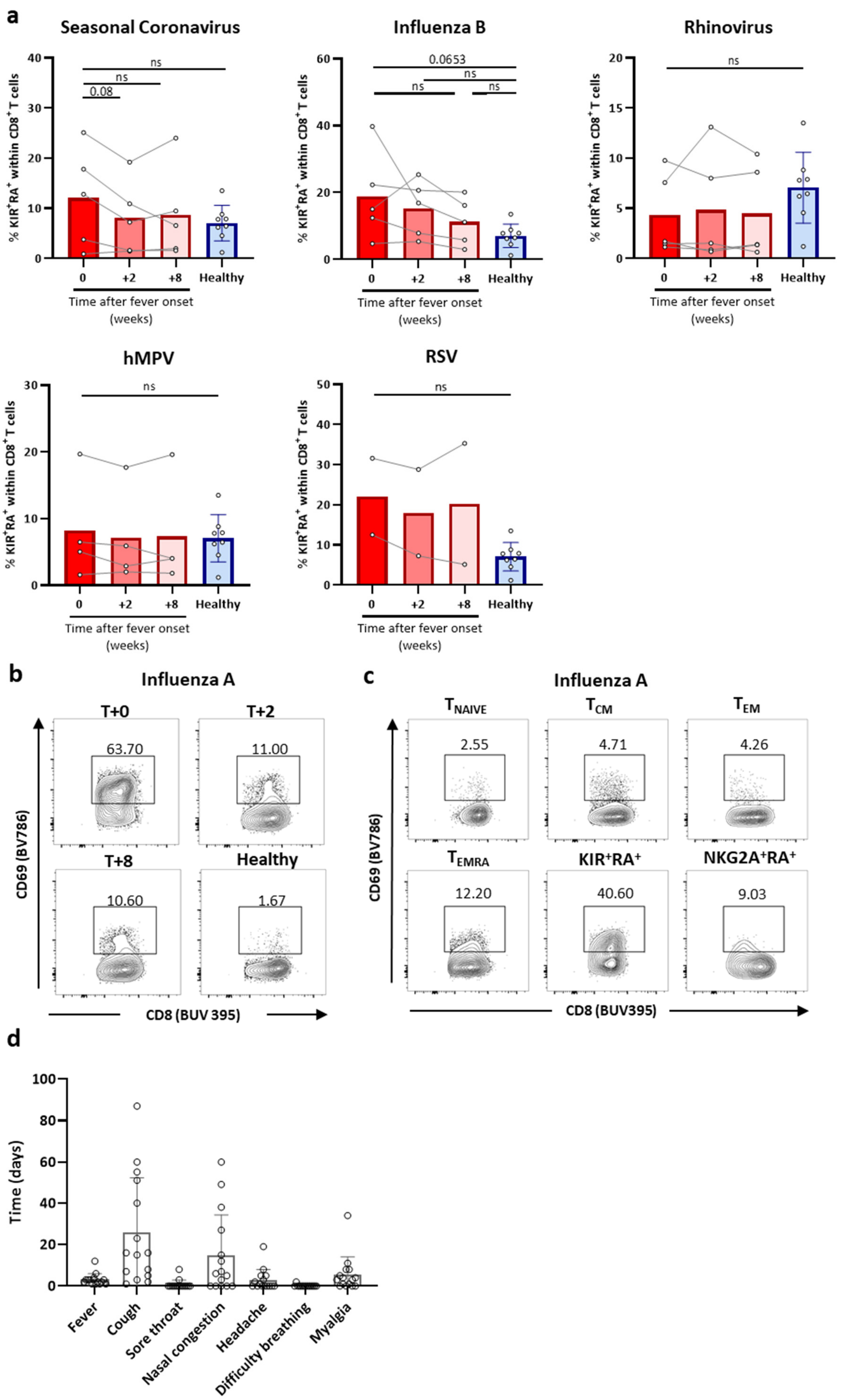
a
Seasonal Coronavirus
Influenza B
Rhinovirus
hMPV
RSV
% KIR+RA+ within CD8+ T cells
Time after fever onset (weeks)
0
+2
+8
Healthy
ns
0.08
0.0653
b
Influenza A
T+0
T+2
T+8
Healthy
63.70
11.00
10.60
1.67
CD69 (BV786)
CD8 (BUV 395)
c
Influenza A
TNAIVE
TCM
TEM
TEMRA
KIR+RA+
NKG2A+RA+
2.55
4.71
4.26
12.20
40.60
9.03
CD8 (BUV395)
d
Time (days)
Fever
Cough
Sore throat
Nasal congestion
Headache
Difficulty breathing
Myalgia

Supplementary Figure 7. Presence of KIR$^+$RA$^+$ T cells during viral respiratory infection.

Blood samples from older adults suffering from a common respiratory virus infection (62-83 years of age, n=36) were analyzed at the acute phase (0) (within two days of fever onset), and during follow-up at +2 and +8 weeks. Healthy asymptomatic individuals (61-82 years of age, n=8) from the same cohort were used as control samples. (**a**) Frequency of KIR$^+$RA$^+$ T cells amongst the CD8$^+$ T-cell population at the three time points in older adults infected with a seasonal coronavirus (n=5), influenza B virus (n=5), Rhinovirus (n=5), human metapneumovirus (hMPV) (n=4), or respiratory syncytial virus (RSV) (n=2). (**b**) Flow cytometry plots showing the proportion of CD69$^+$ cells in KIR$^+$RA$^+$ T cells in influenza A infected older adults at each of the indicated points in time. (**c**) Flow cytometry plots of the proportion of CD69$^+$ cells within each of the indicated CD8$^+$ T-cell subsets. (**d**) Bar graph indicates the symptom duration in days monitored in Influenza-A infected older adults (n=15) from which the symptom score was calculated. Statistical significance of data presented in bar graphs (mean ± s.d.) (**a**) was determined using row-matched one-way ANOVA (with Geisser-Greenhouse correction and Dunnett's post-test) for the difference between the time points and Mann-Whitney U test was used to determine the difference between infected and asymptomatic healthy individuals. (*p <0.05, **p <0.01, or the exact p-value is shown, ns=not significant.)

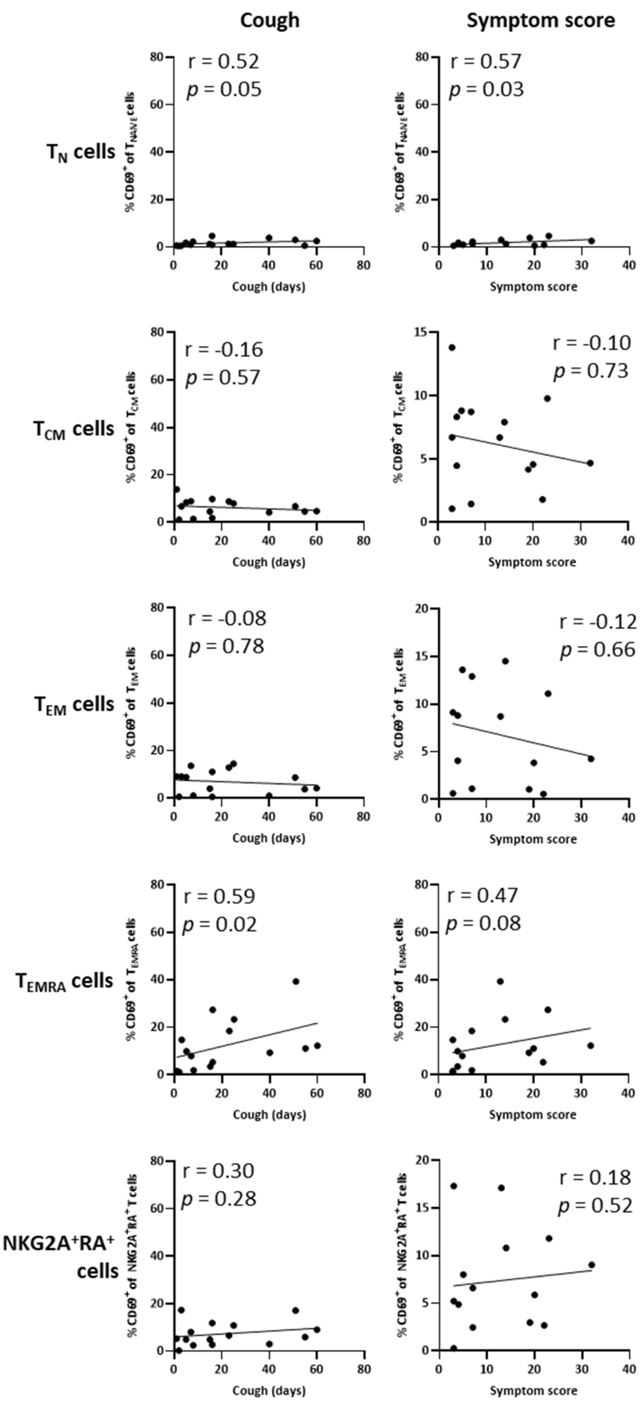

Supplementary Figure 8. Correlations of frequency of activated cells in T-cell subsets at time of symptomatic influenza A infection.

Relationship between the frequency of $CD69^+$ cells within the indicated T-cell subsets and the duration of cough and calculated symptom score in older adults suffering from influenza A virus infection (n=15). Correlations (*r* values) were assessed by Spearman test with the exact *p*-value.

Supplementary Table 1. Samples of older adults with Influenza-like Illness

Respiratory virus detected	Strain	Number	Median Age (years)
**Influenza A virus (n=15)**	H3N2	12	68
	H1N1	3	
**Influenza B virus (n=5)**	Yamagata/16/88	5	63
**Coronavirus (n=5)**	C229E	2	
	HKU1	1	65
	OC43	2	
**Respiratory Syncytial Virus (n=2)**	RSV-A	2	74
**Human metapneumovirus (n=4)**	-	4	71
**Rhinovirus (n=5)**	-	5	73
**Asymptomatic Healthy Controls (n=8)**	None detected	8	70,5

Supplementary Table 2. Samples of adults with COVID-19

Respiratory virus detected	Strain	Number	Median Age (years)
**Coronavirus (n=9)**	SARS-CoV-2	9	43
**Asymptomatic Healthy Controls (n=9)**	Not detected	9	44

Supplementary Table 3. Healthy individuals		
**All individuals (n=50)**		**Age (years)**
	Median Age	52
	Lowest	21
	Highest	82
**Female (n=21)**		
	Median Age	50
	Lowest	31
	Highest	82
**Male (n=27)**		
	Median Age	61,5
	Lowest	21
	Highest	80

Supplementary Table 4. Antibodies used in this study

	Fluorochrome	Clone	Isotype	Manufacturer	Catalogue nr.	Dilution	Whole blood Assay Dilution
**Surface markers**							
CD3	BV421	UCHT1	Mouse IgG1, kappa	Biolegend	300434	1:200	-
CD3	FITC	UCHT1	Mouse IgG1, kappa	Biolegend	300440	1:200	1:100
CD4	PE-Cy7	RPA-T4	Mouse IgG1, kappa	Biolegend	300512	1:100	-
CD4	PerCP-Cy5.5	RPA-T4	Mouse IgG1, kappa	Biolegend	300530	1:200	1:100
CD8a	BB515	RPA-T8	Mouse IgG1, kappa	BD Horizon	564526	1:100	-
CD8a	BUV395	RPA-T8	Mouse IgG1, kappa	BD Horizon	563795	1:100	1:200
CD8a	FITC	RPA-T8	Mouse IgG1, kappa	Biolegend	301050	1:50	-
CD19	BV786	HIB19	Mouse IgG1, kappa	Biolegend	302240	1:100	-
CD27	BV510	O323	Mouse IgG1, kappa	Biolegend	302836	1:50	1:100
CD45	Pacific Blue	HI30	Mouse IgG1, kappa	Biolegend	304029	-	1:400
CD45RA	BV605	HI100	Mouse IgG2b, kappa	Biolegend	304134	1:800	1:400
CD45RA	PerCP-Cy5.5	HI100	Mouse IgG2b, kappa	Biolegend	304122	1:50	-
CD56	PE-Cy7	HCD56	Mouse IgG1, kappa	Biolegend	318318	1:100	-
CD69	BV785	FN50	Mouse IgG1, kappa	Biolegend	310932	1:100	1:100
CD122	PE-Cy7	TU27	Mouse IgG1, kappa	Biolegend	339014	1:50	-
CD226	BV786	DX11	Mouse IgG1, kappa	BD	742497	1:100	-
KIR2D	PE	NKVFS1	Mouse IgG1, kappa	Miltenyi Biotec	130-092-688	1:400	1:400
KIR3DL1	PE	5.133	Mouse IgG1	Miltenyi Biotec	130-095-205	1:25	1:200
NKG2A	APC	REA110	Recombinant human IgG1	Miltenyi Biotec	130-113-563	1:400	1:800
TIGIT	PE-eFluor610	MBSA43	Mouse IgG1, kappa	eBioscience	61-9500-42	1:50	-
Fixable Viability Stain	FVS780	N/A	N/A	BD Horizon	565388	1:2000	-
**Intracellular markers**							
CD3	FITC	UCHT1	Mouse IgG1, kappa	Biolegend	300440	1:200	-
CD107a	PE-Cy7	H4A3	Mouse IgG1, kappa	Biolegend	328618	1:50	-
Helios	PE-Cy7	22F6	Armenian hamster IgG	Biolegend	137236	1:200	-
IFN-gamma	BUV737	4S.B3	Mouse IgG1, kappa	BD	564620	1:50	-
IL-2	BV785	MQ1-17H12	Rat IgG2a, kappa	Biolegend	500348	1:50	-
TNF-alpha	PerCP-Cy5.5	Mab11	Mouse IgG1, kappa	Biolegend	502926	1:50	-
